# Spatial-reprogramming derived GPNMB^+^ macrophages interact with COL6A3^+^ fibroblasts to enhance vascular fibrosis in glioblastoma

**DOI:** 10.1186/s13073-025-01553-2

**Published:** 2025-10-31

**Authors:** Yinfei Du, Xinmiao Long, Xuetong Li, Fan Guan, Wei Gao, Kun Deng, Shiyi Wang, Xiang Lin, Meng Huang, Xiaoling She, Shuai Chen, Minghua Wu

**Affiliations:** 1 The Affiliated Cancer Hospital of Xiangya School of Medicine, Central South University/Hunan Cancer Hospital, Central South University, Changsha, Hunan 410013 China; 2 The Key Laboratory of Carcinogenesis of the Chinese Ministry of Health, The Key Laboratory of Carcinogenesis and Cancer Invasion of the Chinese Ministry of Education, Cancer Research Institute, Central South University, Changsha, Hunan 410078 China; 3 FuRong Laboratory, Changsha, Hunan 410078 China; 4 Department of Neurosurgery, Xiangya Hospital, Central South University, Changsha, 410008 Hunan China; 5 Department of Pathology in Second Xiangya Hospital, Central South University, Changsha, Hunan 410011 China; 6 Xiangya School of Public Health, Central South University, Changsha, Hunan 410013 China

**Keywords:** Tumor-associated fibroblasts, Tumor-associated macrophages, Cancer-associated fibroblasts, Immune checkpoint blockade, Anti-angiogenic agents, Single-cell RNA sequencing

## Abstract

**Background:**

Neoadjuvant therapy plays an important role in the treatment of glioblastoma (GBM), but a considerable proportion of patients remain unresponsive to the combination of immune checkpoint blockade (ICB) and antiangiogenic therapy. Understanding the mechanisms underlying resistance to this treatment and developing novel therapeutic strategies are crucial.

**Methods:**

We integrate extensive single-cell and spatial transcriptomic data to dissect the cellular composition and spatial organization of the GBM tumor microenvironment and validate our findings through experiments such as multiplex immunohistochemistry and atomic force microscopy. We applied 101 machine learning algorithms to evaluate the prognostic and immunological value of COL6A3^+^ tumor-associated fibroblasts (TAFs) and GPNMB^+^ monocyte-derived macrophages (MDMs) in multiple GBM cohorts and immunotherapy cohorts.

**Results:**

We constructed a stromal cell atlas in GBM and identified a distinct subset of COL6A3^+^ TAFs with functional characteristics of matrix fibroblasts. We found that COL6A3^+^ TAFs are significantly enriched in non-responders to neoadjuvant combination therapy. These fibroblasts drive the spatial-reprogramming of anti-tumorigenic MDMs into a pro-tumorigenic phenotype. In turn, these reprogrammed immunosuppressive GPNMB^+^ MDMs promote vascular fibrosis mediated by COL6A3^+^ TAFs through the GPNMB-ITGB5 interaction.

**Conclusions:**

Our findings highlight the critical role of COL6A3^+^ TAFs in regulating MDM function and spatial distribution, as well as their contribution to fibrotic tumor vasculature formation. Additionally, we propose targeting COL6A3^+^ TAFs with cilengitide as a potential therapeutic strategy.

**Supplementary Information:**

The online version contains supplementary material available at 10.1186/s13073-025-01553-2.

## Background

Glioblastoma (GBM) is the most aggressive and incurable primary central nervous system cancer. The standard-of-care treatment includes maximum surgical resection of tumors followed by temozolomide-based chemotherapy and fractionated ionizing radiation, but these approaches showed the limited improvement in clinical outcomes for patients; their median survival time is still less than 15 months [1]. Recent years have seen significant advancements in tumor treatment, particularly with the development of neoadjuvant therapy that focuses on strategically targeting the tumor microenvironment (TME), such as antiangiogenic therapy or immune checkpoint blockade (ICB). However, multiple clinical trials have demonstrated that a significant proportion of GBM patients did not achieve complete or partial radiographic remission after undergoing antiangiogenic therapy [2–4]. Meanwhile, the PD-1 antibody pembrolizumab or the PD-L1 antibody atezolizumab has also exhibited therapeutic benefits in several cancers, except for glioblastoma [5, 6]. The combination of antiangiogenic inhibitors and PD-1 antibodies has shown significant efficacy and good safety in certain types of cancer, such as hepatocellular carcinoma and renal cell carcinoma [7]. Antiangiogenic drugs improve the tumor microenvironment by normalizing tumor blood vessel architecture and function [3] and promote the infiltration and function of immune cells; checkpoint blockers enhance the activity of T cells and further inhibit tumor growth. However, the combination effectiveness is limited in GBM [8], which is due to the unique and complex GBM microenvironment: (1) the immunosuppressive microenvironment shaped by an abundance of tumor-associated macrophages (TAMs) [9, 10]; (2) hypoxia exacerbation, T cell sequestration, or drug delivery issues caused by abnormal tumor vasculature, which is mediated by tumor-associated fibroblasts (TAFs) in the TME [11, 12]. Therefore, it is imperative to explore more optimized combination therapy strategies targeting the GBM microenvironment.

TAMs make up 30–50% of all cells in the TME of GBM and are the most infiltrating immune cells [13]. Approximately 85% of these cells are medullary monocyte-derived macrophages (MDMs), which migrate from the blood, and 15% of brain-resident microglia (MG) develop from the yolk sac during embryogenesis [14, 15]. Single-cell RNA sequencing (scRNA-seq) has overturned the traditional classification of macrophages into pro-inflammatory M1 and anti-inflammatory M2 types and confirmed that a large population of immunosuppressive MDMs exist in gliomas [15]. Therefore, dissecting the functional characteristics and spatial heterogeneity of TAMs in GBM is essential for the rational design of effective therapies. Compelling evidence indicates that the spatial distribution of TAMs in GBM is highly compartmentalized, with distinct subsets of MDMs and microglia exhibiting varying abundances and functional characteristics across regions from the edge to the core [16]. Researchers have shown that most MDMs are located in the tumor core, whereas MG cells are predominantly present in the peritumoral region [17]. Moreover, it had been reported that multiple signals regulate macrophage migration and differentiation in the TME [18]. For example, the CCL2/CCR2 and CSF1/CSF1R signaling axes have been widely reported to mediate the recruitment of macrophages [19–21]. In addition, some cells within the tumor microenvironment also regulate macrophage polarization. For example, fibroblasts induce the differentiation of macrophages into an immunosuppressive phenotype by secreting IL6 and IL34 [22, 23]. Tumor cells can promote M2 macrophage polarization in the TME through the CHI3L1/CD44/PI3K/AKT axis [24]. Furthermore, metabolic factors such as high lactate, low oxygen, and excess lipids can also shape macrophage differentiation [25–29]. Therefore, deciphering the regulatory factors governing the spatial distribution and function of these cell populations is crucial for obtaining a comprehensive understanding of the GBM microenvironment.

Targeting TAFs within the TME is considered a promising strategy to improve the therapeutic effectiveness of ICB in pre-clinical models [30]. However, the absence of fibroblasts in the brain led to the common assumption that TAFs are not present in GBM [31]. ScRNA-seq analysis shows that TAFs have been unequivocally identified in GBM and demonstrated to show pro-tumoral effects [32]. Nevertheless, studies on the functional characteristics of TAFs in GBM are still relatively rare. The reciprocal interactions between TAFs and TAMs constitute a critical signaling network within the TME, which is intricately linked to the efficacy of therapeutic interventions. Many studies have substantiated the crucial role of TAFs in modulating TAM M2 polarization within the TME [22]. Furthermore, TAMs have been reported to influence the functional activity of TAFs [12]. Therefore, an in-depth analysis of the interaction between TAFs and TAMs in GBM and the identification of therapeutic strategies are urgently needed.

In this study, we integrated extensive scRNA-seq and spatial transcriptomics data to investigate the mechanisms underlying the failure of antiangiogenic therapy combined with PD-1 inhibitor therapy. We constructed a stromal cell atlas in GBM and reported that the proportion of COL6A3^+^ TAFs significantly increased in non-responders to neoadjuvant therapy. COL6A3^+^ TAFs mediated the spatial-reprogramming of MDM subpopulations to enhance vascular fibrosis and lead to T cell exclusion, limiting the efficacy of neoadjuvant combination therapy. Finally, we propose that targeting specific GBM-associated TAFs possessed precise therapeutic potential in this context.

## Methods

### Primary GBM-associated TAFs isolated from patient samples

Primary TAFs and GBM tumor cells were isolated via the method reported by Jain et al. [32]. We used a sequential trypsin digestion method, in which dissociated GBM samples were cultured at 37 °C in StemGro MSCplus Basic Culture Medium (T310KJ, BasalMedia) under a humidified atmosphere of 5% CO2/95% air. The cells were continuously digested with 0.25% trypsin–EDTA. Since primary tumor cells have lower adhesion than TAFs do, we performed trypsinization for 30 s and discarded the supernatant to remove the tumor cells with lower adhesion, leaving the TAFs behind. Then, we performed 10-min trypsin digestion to isolate the TAFs, which were subsequently transferred to a new plate.

### Patient-derived glioblastoma organoid

The method used is consistent with that reported by Jacob et al. [33]. We cut the resected tumor tissue into small pieces approximately 0.5–1 mm in diameter, transferred them to ultra-low attachment six-well plates containing GBO medium, and cultured them on an orbital shaker at 120 rpm under 5% CO_2_ at 37 °C. GBOs were exposed to 1% O_2_ for 2 days to simulate hypoxic conditions. The patient information for organoids used in this study is shown in Table S2 (Additional file 2).

### Human peripheral blood mononuclear cell-derived T cells

Fresh anticoagulated blood is mixed with a sample diluent and slowly layered onto the separation medium, followed by gradient centrifugation. After centrifugation, the white cell layer at the interface is carefully collected to obtain high-purity PBMCs. The cell pellet was resuspended in RPMI-1640 medium (ZQXZBIO, ZQ-200) supplemented with 10% FBS, penicillin–streptomycin, and cytokines including IL-2 (primegene, 600 U/mL), IL-7 (primegene, 5 ng/mL), and IL-15 (primegene, 5 ng/mL). Primary T cells were stimulated with anti-CD28 (Thermo Fisher, 1 µg/mL) and anti-CD3 (Thermo Fisher, 100 ng/mL) antibodies.

### Cell lines and cell culture

The human monocytic leukemia cell line (THP-1) was obtained from Prof. Juanjuan Xiang (Central South University, China).

All the cells were cultured under standard atmospheric conditions of 5% CO_2_ at 37 °C. Primary tumor cells were cultured in Dulbecco’s Modified Eagle’s Medium (DMEM, Gibco) supplemented with 10% fetal bovine serum (FBS, ExCell Bio), penicillin, and streptomycin (100 μg/mL). GBM-derived primary TAFs were cultured in StemGro Mesenchymal Stem Cell (MSC) Expansion Medium (BasalMedia). Patient-derived primary MDMs and THP-1 cells were cultured in RPMI 1640 supplemented with 10% FBS (ExCell Bio) and 1% penicillin–streptomycin (Solarbio). The treatment process for THP-1 cells is as follows: THP-1 cells were induced to differentiate into macrophages by culture with 100 ng/mL phorbol 12-myristate 13-acetate (PMA, MCE) for 24 h. The cells were subsequently treated with 100 ng/mL LPS (MCE) and 20 ng/mL IFN-γ (MCE) for 48 h to induce polarization into ICAM1^+^ MDMs. Additionally, in the control group, COL6A3^+^ TAF supernatant was added to induce the cells to differentiate into GPNMB^+^ MDM. In the different treatment groups, sotuletinib (5 μM) and a TGFβ3 antibody (100 ng/mL) were added to evaluate the blocking effect (Additional file 1: Fig. S8A). The treatment process for COL6A3^+^ TAFs is as follows: after primary cells were cultured for 7 days, the medium was replaced, and the supernatant was retained. For the experimental groups, sGPNMB (MCE) and cilengitide (MCE) were added at concentrations of 100 ng/mL and 10 μM, respectively. The oxygen concentration in the hypoxic environment was maintained at 1% O_2_.

### Immunofluorescence

The cultured cell slides were fixed in 4% paraformaldehyde for 15 min, blocked with bovine serum albumin (BSA, MeilunBio) for 30 min at 37 °C, and then incubated overnight at 4 °C with primary antibodies, including anti-human ICAM1, anti-human GPNMB, anti-human COL6A3, and anti-human PDGFRA. The slides were then incubated with secondary antibodies conjugated to Alexa Fluor 488 and 647 at 37 °C for 1.5 h. Afterward, the samples were counterstained with 4′−6-diamidino-2-phenylindole (DAPI, Servicebio) for 10 min and mounted with colorless nail polish. Confocal imaging was performed using a Nikon-CSU-W1 spinning disk equipped with a microlens SoRa emission disk. Images were captured on an inverted Nikon Eclipse Ti2 microscope (Nikon Instruments) connected to the Yokogawa motorized spinning disk system (CSU-W1 SoRa, Yokogawa Electric).

### Multiplex immunohistochemical staining

Multiplex immunohistochemistry (mIHC) was performed on formalin-fixed, paraffin-embedded (FFPE) slides of human GBM tissue. In brief, 3-μm-thick sections were cut from paraffin blocks and mounted on slides. The paraffin sections were then deparaffinized with xylene and rehydrated with ethanol. Antigen retrieval was performed via microwave heat treatment. After preincubation with blocking buffer at room temperature for 10 min, the sections were sequentially incubated with primary antibodies, including anti-human GPNMB (Proteintech) and anti-human ITGB5 (ProMabBiotechnologies Inc.) (Additional file 4: Resource List). The slides were then incubated with horseradish peroxidase (HRP)-conjugated secondary antibodies, followed by tyramide signal amplification (TSA). Microwave treatment was applied between each TSA step. After all the target antigens were labeled, the cell nuclei were stained with 4′−6-diamidino-2-phenylindole (DAPI, Servicebio). Multispectral images were obtained by scanning stained slides in the range of fluorescence excitation spectra (420–720 nm) via a Mantra system (CaseViewer).

### Flow cytometry

To conduct surface staining, we mixed the appropriate antibodies with the cells at room temperature for 30 min and washed them with 1% BSA/PBS. Zombie Aqua dye was used to exclude dead cells. For cytokine staining, we first treated the cells with a protein transport inhibitor (containing brefeldin A) for 6 h. Subsequently, intracellular staining was performed at 4 °C for 30 min via permeabilization buffer and appropriate antibodies, as previously described. During flow cytometric sorting and analysis, CD45^+^CD11b^+^ICAM1^+^GPNMB⁻ cells were defined as ICAM1^+^ MDMs, whereas CD45^+^CD11b^+^ICAM1⁻GPNMB^+^ cells were defined as GPNMB^+^ MDMs. The antibodies used for flow cytometry are listed in the resource list (Additional file 4: Resource List).

#### ELISA

The concentrations of CSF1 and TGFβ3 in the supernatants of COL6A3^+^ TAFs and human primary tumor cells were quantified via human CSF1 and TGFβ3 ELISA kits (EH0014, FineTest; JL20084-96 T, Shanghai9Jonlnbio Industrial Co., Ltd.) following the manufacturer’s instructions.

### Chemotaxis assay

MDMs were seeded at a density of 5 × 10^4^ cells per well in the upper chamber of a Transwell insert with an 8-μm pore-size polycarbonate membrane, while the lower chamber was filled with the supernatant from COL6A3^+^ TAFs. Sotuletinib (5 μM) and TGFβ3 (100 ng/mL) antibodies were added to the upper chamber. After incubation at 37 °C for 24 h, the cells were fixed in 10% paraformaldehyde for 30 min and stained with 0.1% crystal violet (Abiowell) for another 30 min. Finally, the number of migrated cells at the bottom of the membrane was assessed by counting three randomly selected fields.

### RT-qPCR

Total mRNA was isolated from cells via TRIzol reagent (Adilab), and cDNA was synthesized from total RNA by All-in-One First-Strand Synthesis Master Mix (BestEnzymes Biotech). Its concentration and purity were assessed with a Nanodrop2000 microultraviolet spectrophotometer. qPCR analysis was then performed on a Bio-Rad real-time PCR system (Bio-Rad, Hercules, CA, USA; 788BR06968). GAPDH served as the internal control. Three independent experiments were conducted, and relative mRNA expression levels were determined via the 2^−ΔΔCt^ method (ΔΔCt = ΔCt [case] – ΔCt [control]). The sequences of primers used for RT-qPCR are listed in Table S6 (Additional file 2).

### Atomic force microscopy measurement

The cells seeded on the slides were fixed with a 0.5% glutaraldehyde solution for 1 min. After the glutaraldehyde solution was removed, the cells were washed with PBS for 5 min, and this process was repeated three times. In the final step, after removing the buffer, the dish was dried at room temperature. An MFP-3D-BIO AFM (ASYLUM RESEARCH) was used to evaluate the elastic behavior of the treated cells. The AFM, mounted on an inverted optical microscope, was installed on a camera. A V-shaped highly sensitive probe (MLCT-D, BRUKER) with a nominal spring constant of 0.01–0.06 N/m, a radius of 20 nm, and a nominal frequency of 10–20 kHz was mounted on the holder. The Hertz model was employed to fit the Young’s modulus. Imaging was performed in “AC mode (Tapping mode)” with an imaging frequency of 1 Hz, using the same probe type as that used for the Young’s modulus measurements. All 3D visualizations were created via IGOR PRO 19.06.65 software.

### Datasets analyzed in this study

We have collected and analyzed published scRNA-seq, bulk RNA-seq, and spatial transcriptomics datasets. These datasets and their analyses are described below. For the datasets involving single-cell analysis, we collected data from the Gene Expression Omnibus (GEO) database (https://www.ncbi.nlm.nih.gov/geo/) [34] and the National Genomics Data Center of China (https://ngdc.cncb.ac.cn/) [35]. We collected data from 33 newly diagnosed GBM patients (GSE131928, GSE138794, GSE182109, OMIX003593, HRA007138) [9, 36–40], 6 newly diagnosed low-grade glioma (LGG) patients (GSE182109, GSE138794) [36, 37], 10 recurrent GBM patients (GSE182109, OMIX003593) [9, 36], 7 GBM patients with a positive response to neoadjuvant therapy (OMIX003593), and 5 GBM patients with no response to neoadjuvant therapy (OMIX003593) [9]. The neoadjuvant therapy involved a combination of anlotinib (tyrosine kinase inhibitor) and pembrolizumab (PD-1 inhibitor). For the spatial transcriptomics data, we collected sequencing data of 2 newly diagnosed GBM patients and 1 GBM patient with no response to neoadjuvant therapy from OMIX003593 [9]. For survival analysis, deconvolution algorithms, and machine learning models built from bulk RNA-seq cohorts, we used data from 691 glioma patients downloaded from the Cancer Genome Atlas (TCGA) database (http://gdac.broadinstitute.org/) [41], 693 and 325 glioma patients from the Chinese Glioma Genome Atlas (CGGA) database (https://www.cgga.org.cn/) [42], 270 glioma patients from Ivy GBM Atlas Project (Ivy-GAP, http://glioblastoma.alleninstitute.org/) [43], 371 glioma patients from the Glioma Longitudinal AnalySiS (GLASS) database (http://www.synapse.org/glass) [44], and 487 glioma patients obtained through microarray chip sequencing (GSE108474) [45]. For the bulk RNA-seq cohorts used to predict immunotherapy efficacy, we used data from 234 renal cell carcinoma patients treated with PD-1 blockade [46], data from 85 melanoma patients treated with nivolumab [47], data from 311 bladder cancer patients treated with atezolizumab (Imvigor210) [48], and data from 34 GBM patients treated with nivolumab (PRJNA482620) [49]. The clinical information for all patients involved in single-cell sequencing analysis is summarized in Table S1 (Additional file 2).

### Preprocessing of scRNA-seq data

The scRNA-seq data preprocessing was carried out with the Seurat package (v.4.4.0) [50] in R. Initially, RNA background contamination was removed via decontX (v.1.0.0) [51], and cells were filtered on the basis of gene counts of less than 200, as well as the top 2% of genes and unique molecular identifier (UMI) counts. Low-quality cells with mitochondrial gene expression exceeding 25%, ribosomal gene expression below 3%, and hemoglobin gene expression above 1% were excluded. Doublets were identified and excluded via DoubletFinder (v.2.0.3) [52]. To reduce the dimensionality of the data, principal component analysis was conducted on the top 2000 most variable genes in the dataset. Batch effects across patient samples were corrected by applying the Harmony R package (v.1.2.0) [53] to the principal components. The FindNeighbors function was used to identify the nearest neighbors for graph-based clustering, utilizing the first 50 principal components. Cluster identification was achieved via the FindCluster function with resolutions ranging from 0.1 to 1. Cell visualization was done using Uniform Manifold Approximation and Projection (UMAP) and t-distributed stochastic neighbor embedding (tSNE). Differentially expressed genes for each cluster were determined via the FindAllMarkers function (min.pct = 0.2 and logfc.threshold = 0.2).

### Inference of CNV

Copy number variations (CNVs) were estimated via the inferCNV (v.1.10.1) package [54]. Myeloid cells and NK/T cells were used as the baselines to estimate CNVs for the remaining cell types. Hierarchical clustering was applied to distinguish nonmalignant cells from malignant cells, which exhibited distinct chromosomal deletions or amplifications.

### Pathway activity and enrichment analysis

We scored the cells with known gene signatures via the AddModuleScore function. To explore the functions of the differentially expressed genes (DEGs), we analyzed the human C2 and C5 gene sets from MSigDB via the msigdbr R package (v.7.5.1) and the “clusterProfiler” R package (v.4.12.6) [55]. Pathways with adjusted *p* values less than 0.05 were considered significantly enriched. We also performed GSVA analysis on the cell count matrix via GSVA (v.1.52.3) [56]. Additionally, gene set enrichment analysis (GSEA) was used to compare the differentially expressed genes between the two groups [57]. GSEA uses the two-sided permutation test with Benjamini–Hochberg adjustment. A normalized enrichment score (NES) > 1 and a corrected *p* value < 0.05 were considered significant.

### Cellular interaction analysis

We employed the R packages CellChat (v.1.6.1) [58] and CellPhoneDB (v.2.1.1) [59] to infer and analyze intercellular communication networks quantitatively on the basis of our scRNA-seq data. The prediction of enriched tor-ligand pairs between two cell types was performed on the basis of receptor expression in one cell type and the corresponding ligand expression in the other cell type. We considered only those ligands and receptors that were expressed in at least 15% of the cells within a given cell type. Visualization was performed via the ktplots R package (v.2.4.0). Additionally, we used the Nichenet package (v.2.2.0) [60] to infer interactions between COL6A3^+^ TAFs and MDMs. We extracted the top 20 ligands and the top 100 target genes from the genes differentially expressed between the “sending cells” and “receiving cells” for receptor-ligand activity analysis. The expression of the differentially expressed ligands and receptors was calculated based on the average gene expression in each cell type and visualized via heatmaps, with normalization applied across different subtypes.

### Trajectory analysis

We used the Slingshot R package (v.2.12.0) for trajectory inference [61]. Slingshot employs a sequential principal curve algorithm, which constructs smooth curves from the minimum spanning tree to calculate pseudotime. Simultaneously, the tradeSeq algorithm was applied to identify DEGs across pseudotime intervals, revealing that gene expression patterns are influenced by branching fates. The Monocle 2 algorithm (v.2.32.0) [62] was subsequently used for validation, where the DDRTree method in reverse graph embedding was applied to construct the pseudotime tree, with the starting point adjusted according to the biological context.

### Cell deconvolution for bulk RNA-seq datasets

We used the online tool CIBERSORTx algorithm to perform cell-type deconvolution, estimating the proportions of different cell subpopulations across various samples [63]. Our annotated scRNA-seq glioma dataset was used as the single-cell reference matrix, combined with public bulk RNA-seq and microarray datasets for analysis. The inputs included the single-cell reference matrix and bulk RNA-seq data, with other parameters set to the algorithm’s default options. The signature genes used for deconvolution are automatically generated by the algorithm (Additional file 3: Table S7).

### 10 machine learning algorithms to construct predictive models

We used the Mime1 R package (v.0.0.0.90) [64] for prognostic gene selection and model construction. First, we performed univariate Cox regression on the provided genes in the training dataset to screen for prognostic variables. Then, we combined 10 machine learning algorithms and their 101 unique combinations. These algorithms include random survival forest (RSF), least absolute shrinkage and selection operator (Lasso), elastic net (Enet), ridge, CoxBoost, stepwise Cox, survival support vector machine (survival-SVM), generalized boosted regression modeling (GBM), supervised principal component analysis (SuperPC), and Cox partial least squares regression (plsRcox). The area under the ROC curve (AUC) was calculated for both the training and validation sets. The optimal model was determined by identifying the model with the highest average time-dependent AUC value in both the training and validation sets.

### Survival analysis and prediction of immunotherapy response

We performed survival analysis via the R package survival (V.3.5–8). Patients were grouped based on GSVA scores for specific gene signatures. The Cox proportional hazards model was used to calculate hazard ratios (HRs) and 95% confidence intervals (CIs). The survfit function was employed to model Kaplan–Meier survival curves, which were analyzed via a two-sided log-rank test. We also used the TIDE algorithm [65] to evaluate the relationship between cell infiltration levels and TIDE scores, thereby reflecting the effectiveness of immunotherapy.

### Spatial transcriptomics data analysis

Spatial transcriptomics data were analyzed in R using the Seurat (V.4.4.0) package following the recommended data processing guidelines [50]. Genes expressed in fewer than three spots and genes with a total count of less than 200 were filtered out. After normalizing across spots using the SCTransform function, dimensionality reduction was performed via PCA and UMAP. Clustering was conducted with the default resolution using the top 30 principal components. The gene sets derived from Ivy-GAP, associated with glioblastoma anatomical features, were used to infer the pathological locations of spots on spatial transcriptomic sections (CT, cellular tumor; LE, leading edge; MVP, microvascular proliferation; PAN, pseudopalisading cells around necrosis). The AddModuleScore function was used to assess the pathological location score for each spot, and the highest score was used for annotation. Additionally, this function was applied to compute scores for the top 30 feature gene signatures of different cell subtypes for each spot while minimizing the inclusion of tumor-specific genes (average expression < 0.25 in tumor cells) in the scoring process.

### Spatial trajectory inference

We utilized the SPATA2 package [66] to investigate the dynamic biological processes occurring from the MVP region to the PAN region at spatial resolution. We converted the Seurat object into a Spata object using the asSPATA2 function. Next, we employed the createSpatialTrajectories function to generate a spatial trajectory starting from a vessel-enriched high-density area and ending at a pseudopalisading necrosis high-density region. The trajectory inference was performed using a step size of 15 pixels and is visualized in yellow on the surface plot. The plotSpatialTrajectories function was then used to visualize the changes in cell proportions along the trajectory.

### Bulk RNA-seq analysis of COL6A3^+^ TAFs

Total RNA from COL6A3^+^ TAFs in both the control and experimental groups was extracted using Trizol reagent (Sangon Biotech). RNA libraries were constructed and sequenced on the Illumina platform (Sangon Biotech). The quality of the raw sequencing reads was checked via FASTQ, and DESeq2 (v.1.44.0) was used for count normalization and analysis [67]. Genes with log2 fold-change > 1 and adjusted *p*-values < 0.05 were considered DEGs and then converted to Entrez gene IDs. Enrichment analysis was performed using the clusterProfiler (V.4.12.6) R package [55].

### Statistical analysis

All the statistical analyses and data presentations were performed using R (v.4.4.0) and GraphPad Prism 9.5. Two-group continuous variable data were analyzed using a two-tailed unpaired *t*-test or paired Student’s *t*-test, while multiple groups were compared using one-way ANOVA with Tukey’s test or two-sided unpaired Wilcoxon test. Survival analysis was performed using the Cox regression model or Kaplan–Meier method, with comparisons made using the log-rank test. A *p*-value < 0.05 was considered significant (*p < 0.05; **p < 0.01; ***p < 0.001; ****p < 0.0001). The data are presented as the means ± standard deviations (SDs). In the boxplots, the central line indicates the median, the box edges correspond to the upper and lower quartiles, and the whiskers extend up to 1.5 times the interquartile range. Unless otherwise specified, all experiments were conducted at least three times with biologically independent samples.

## Results

### The stromal cell atlas in GBM

In normal brain tissue, there are no “stromal cells” in the traditional sense, but there is the presence of extracellular matrix (ECM) and some cells with functions similar to those of matrix cells. To investigate the stromal cell landscape in GBM, we analyzed scRNA-seq data from tumor specimens from 61 glioma patients, including newly diagnosed GBM patients (*n* = 33) [9, 36–39], newly diagnosed low-grade glioma (LGG) patients (*n* = 6) [36, 37], recurrent GBM patients (*n* = 10) [36], neoadjuvant-responder GBM patients (*n* = 7) [9], and neoadjuvant-nonresponder GBM patients (*n* = 5) [9] (Additional file 1: Fig. S1 and Additional file 2: Table S1). The neoadjuvant therapy is a combination of pembrolizumab (PD-1 inhibitor) and anlotinib (tyrosine kinase inhibitor). Patients whose tumor volume remained stable or who continued to shrink for at least 3 months were classified as responders. A total of 381,277 cells were subjected to stringent quality control, including the removal of background RNA contamination and doublet cells. These cells were then annotated into seven major cell populations based on classical single-cell marker expression and copy number variations (CNVs), with batch effects corrected using the Harmony algorithm (Additional file 1: Fig. S2A–S2D and Additional file 2: Table S3). The cell composition and infiltration fraction of these seven major cell types showed significant heterogeneity across different patient groups (Additional file 1: Fig. S2E–S2F). The number of NK/T cells was significantly increased in patients receiving neoadjuvant combination therapy, which is consistent with previous findings [68]. Interestingly, the proportion of stromal cells significantly increased in patients receiving neoadjuvant combination therapy, potentially revealing previously unrecognized stromal cells in this treatment context (Additional file 1: Fig. S2E–S2F). We further performed subgroup annotation of oligodendrocytes via widely recognized marker genes [69, 70], which identified astrocytes and oligodendrocyte progenitor cells, thereby validating the reliability of our dataset (Additional file 1: Fig. S2G–S2H and Additional file 2: Table S3).

**Fig. 1:**
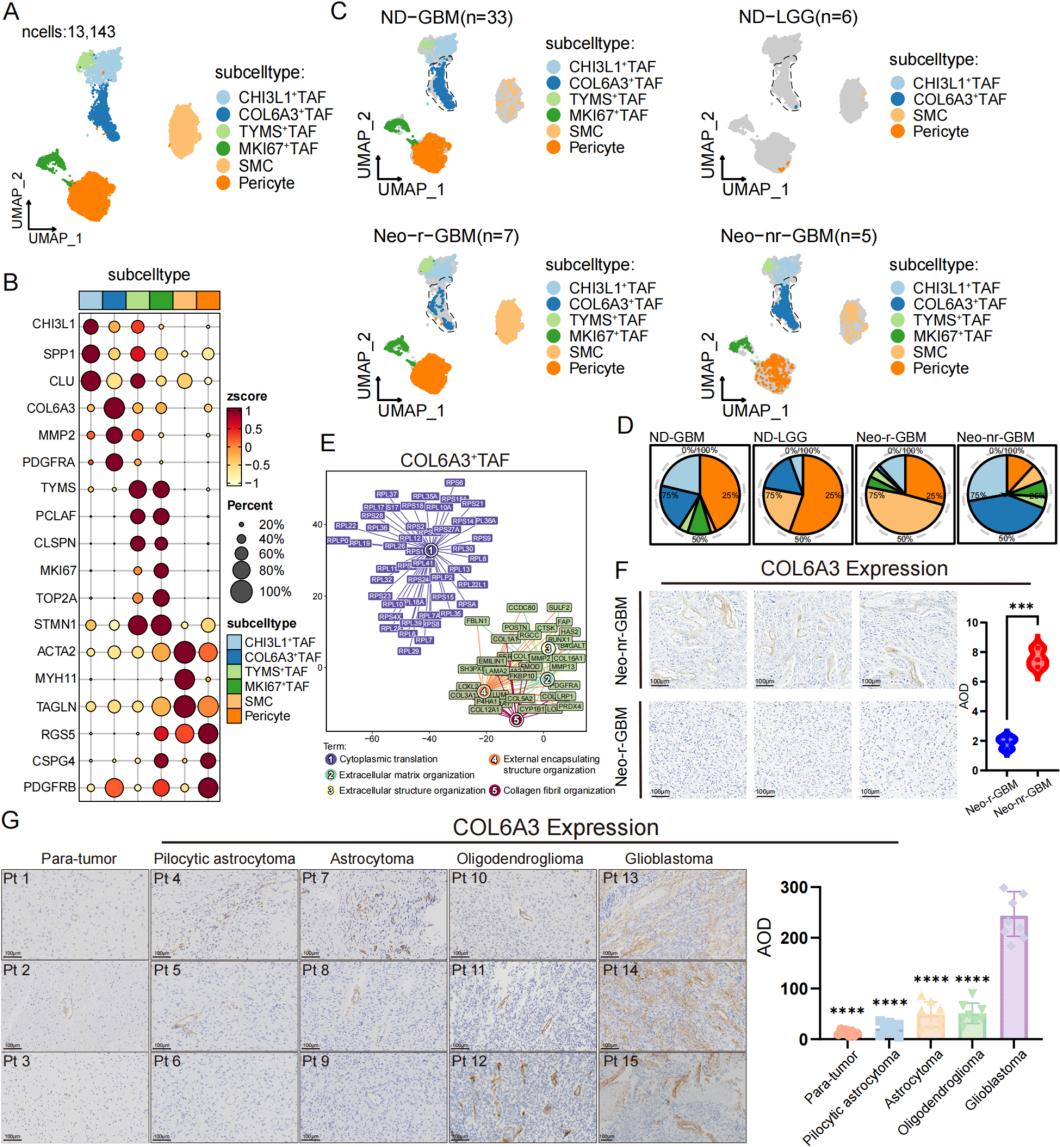
A significant reduction of COL6A3^+^ TAFs in neoadjuvant responders. **A** Uniform manifold approximation and projection (UMAP) plot showing different subtypes of stromal cells (*n* = 13,143 cells). **B** Dot plot showing the top 3 highly expressed genes for each subtype of stromal cells. **C** UMAP plot showing the relative abundance of subtypes based on the sample origin. **D** Pie charts displaying the frequencies of cell subtypes in different patient groups. **E** Gene network diagram showing functional analysis of COL6A3^+^ TAF characterized genes. **F** Representative immunohistochemical staining of pathological slides and box plots showing increasing COL6A3 expression and average optical density (AOD) in responder (*n* = 3) and nonresponder (*n* = 3) samples. **G** Representative immunohistochemical images and AOD quantification of COL6A3 expression in peritumoral regions (normal brain, *n* = 8), pilocytic astrocytoma (WHO grade I, *n* = 8), astrocytoma (WHO grade II/III, *n* = 8), oligodendroglioma (WHO grade II/III, *n* = 8), and glioblastoma (WHO grade IV, *n* = 8)

We further stratified the 13,143 stromal cells into 6 subsets based on representative gene signatures to provide an unbiased characterization of the single-cell landscape of stromal cells in GBM (Fig. 1A, Additional file 1: Fig.S3A and Additional file 2: Table S4). Specifically, the CHI3L1^+^ TAF cluster exhibited antigen presentation and leukocyte chemotaxis response programs characterized by the upregulation of genes (CHI3L1, SPP1, CLU, HLA-DPA1, CD74, and MDK), along with a stress response to metal ions and features related to gliogenesis. The COL6A3^+^ TAF cluster was characterized by processes involved in extracellular matrix organization and collagen fibril organization. Interestingly, these cells also displayed features of negative regulation of cell motility. The TYMS^+^ TAF and MKI67^+^ TAF clusters presented increased expression of cell proliferation-related functional modules, with TYMS^+^ TAFs also exhibiting features related to plasminogen activity. The smooth muscle cell (SMC) cluster was characterized by the upregulation of muscle cell functional programs and the expression of signature genes (ACTA2, MYH11, and TAGLN). The pericytes displayed functional features related to maintaining blood vessel diameter and highly expressed signature genes (RGS5, CSPG4, and PDGFRB) (Fig. 1B and Additional file 1: Fig. S3A–B). None of these stromal cells showed CNV changes, further distinguishing them from tumor cells (Additional file 1: Fig. S2D).

### COL6A3^+^ TAFs are the most decrease in neoadjuvant responders

Analyzing cell infiltration proportions within the TME of patients with varying therapeutic outcomes through a single-cell perspective offers a higher-dimensional understanding of the critical cellular subpopulations driving treatment efficacy. By analyzing the infiltration of stromal cells in different GBM patients, we found that compared with those in newly diagnosed GBM patients or neoadjuvant non-responders, the cells that decreased the most were COL6A3^+^ TAFs in neoadjuvant responders. COL6A3^+^ TAFs were characterized by high expression of collagen genes such as COL6A3, COL3A1, and COL1A1, as well as genes involved in extracellular matrix component regulation, including MMP2, CTHRC1, and SFRP4 (Fig. 1B and Additional file 1: Fig. S3B). Moreover, SMCs and pericytes predominated in neoadjuvant therapy patients, indicating a relatively normal vascular system (Fig. 1C–D and Additional file 1: Fig. S3C–E). Gene Ontology (GO) functional enrichment analysis revealed enhanced activities in cytoplasmic translation, extracellular matrix organization, extracellular structure organization, external encapsulating structure organization, and collagen fibril organization in COL6A3^+^ TAFs (Fig. 1E). This reflects their characteristics as matrix fibroblasts. Immunohistochemical analysis confirmed that COL6A3 expression was significantly elevated in neoadjuvant non-responders compared with neoadjuvant responders (Fig. 1F). We further validated COL6A3 expression at the major cell type level via integrated single-cell data, and the results revealed that high expression of COL6A3 occurred only in stromal cells, suggesting that COL6A3 can serve as a unique marker for COL6A3^+^ TAFs (Additional file 1: Fig. S3F). In addition, to demonstrate that COL6A3 expression is restricted to GBM, we analyzed the scRNA-seq data of normal brain tissue stromal cells published by Bejarano et al. [71]. The results revealed that COL6A3 was nearly not expressed in normal brain tissue (Additional file 1: Fig. S3G). We next performed survival analysis on the top 30 signature genes of COL6A3^+^ TAFs via the Cancer Genome Atlas (TCGA)-GBM/LGG RNA sequencing (RNA-seq) dataset and the Chinese Glioma Genome Atlas (CGGA)-GBM/LGG RNA-seq dataset. We classified patient clinical data into three categories, glioblastoma, astrocytoma, and oligodendroglioma, on the basis of the updated WHO 2021 criteria for GBM. Kaplan–Meier survival curves demonstrated that increased infiltration of COL6A3^+^ TAFs was associated with shorter overall survival in GBM patients (Additional file 1: Fig. S4A–B). Furthermore, we utilized deconvolution analysis to estimate the proportions of different stromal cell and major cell populations in the TCGA-GBM/LGG RNA-seq dataset and CGGA-GBM/LGG RNA-seq dataset. Consistently, our analysis revealed that the proportion of infiltrating COL6A3^+^ TAFs was significantly higher in GBM compared to LGG. Additionally, patients with IDH wild-type tumors presented a greater proportion of COL6A3^+^ TAFs than did those with IDH mutations, whereas patients with an unmethylated MGMT promoter also displayed a higher proportion of COL6A3^+^ TAFs (Additional file 1: Fig. S4C–D). Finally, we collected tumor samples classified according to the WHO 2021 criteria for GBM and performed immunohistochemical validation. The results showed that COL6A3 is highly expressed in GBM, while its expression is much lower in lower-grade gliomas and normal brain tissue (Fig. 1G). These findings suggest that COL6A3^+^ TAFs potently serve as indicators of GBM progression. Taken together, these data indicate that COL6A3^+^ TAFs, with their unique extracellular matrix regulatory functions, significantly influence the prognostic outcomes of GBM patients and are a contributing factor to the limited efficacy of neoadjuvant therapy in GBM patients.

### Identification of two MDM subtypes exhibiting opposite prognostic characteristics through distinct T cell activation capacities

To comprehensively determine the heterogeneity of myeloid cells in GBM, we divided myeloid cells into four types based on established markers (Additional file 1: Fig. S5A–B and Additional file 2: Table S3). Analyzing the distribution proportions of different types of myeloid cells, we found that MDM infiltration was greater in GBM compared to LGG, whereas MG showed the opposite trend, which is consistent with previous reports [72] (Additional file 1: Fig. S5C).

**Fig. 2:**
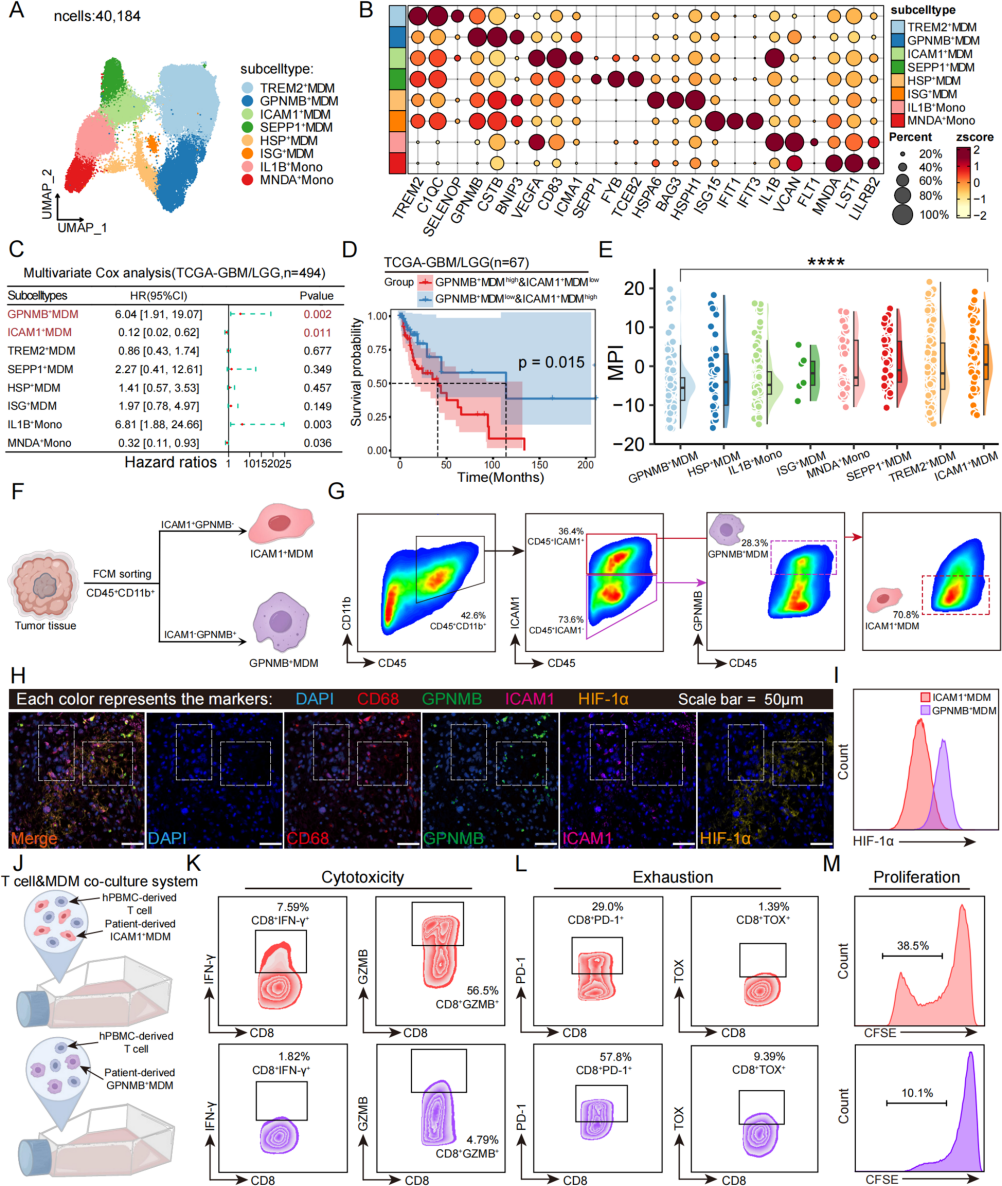
Assessment of the degree of hypoxia and T cell activation function in two MDM subpopulations with opposite prognostic characteristics. **A** UMAP plot showing different subtypes of MDMs (*n* = 40,184 cells). **B** Dot plot showing the top 3 highly expressed genes for each subtype of MDMs. **C** Forest plot showing the results of multivariate cox regression analysis of overall survival in patients in the TCGA-GBM/LGG cohort (*n* = 494), with error bars showing 95% confidence intervals. **D** Kaplan–Meier plot showing that a lower proportion of ICAM1^+^MDMs and a higher proportion of GPNMB^+^MDMs based on GSVA scores grouping is associated with shorter overall survival. **E** Raincloud plots displaying the MPI scores of different MDM subgroups. **F** Experimental design of isolating patient-derived ICAM1^+^ MDMs and GPNMB^+^ MDMs. **G** Representative FCM plots showing the percentages of patient-derived ICAM1^+^ MDM and GPNMB^+^ MDM. **H** Representative mIHC images of DAPI, CD68, GPNMB, ICAM1, and HIF-1α. Scale bar = 50 μm. The left dashed box shows ICAM1^+^ MDM staining, and the right box shows GPNMB^+^ MDM staining. **I** Ridge plot showing the expression level of HIF-1α. **J** Experimental design of the hPBMCs-derived T cells and primary MDM co-culture system. **K** Representative FCM scatterplots showing the T cell cytotoxic activity level. **L** Representative FCM scatterplots showing the T cell exhaustion level. **M** Representative FCM scatterplots showing the T cell proliferation level

MDMs are the primary determinants shaping the distinct intratumoral immune landscape in GBM and contribute to tumorigenesis and therapeutic resistance [73]. A total of 40,184 cells were further classified into eight subpopulations based on previously reported marker genes (Fig. 2A). Specifically, the TREM2^+^ MDM cluster contained the largest number of cells. These cells exhibited the upregulation of TREM2, C1QC, and SELENOP, which primarily participate in phagocytosis and antigen presentation and mirrored the functional features of SEPP1^+^ MDM. The GPNMB^+^ MDM cluster displayed a prominent hypoxia response program characterized by the upregulation of hypoxia response genes (GPNMB, BNIP3, and CSTB). The ICAM1^+^ MDM cluster was characterized by high expression of inflammatory- and angiogenesis-related genes (IL1B, TNF-α, VEGFA, and ICAM1). The ISG^+^ MDM cluster showed upregulation of IFN-stimulated genes (IFIT1, IFIT3, and ISG15). Lastly, the HSP^+^ MDM cluster was enriched for stress-related genes (HSPH1, HSPA6, and BAG3), which are potentially involved in cellular stress response programs. Additionally, we identified two distinct monocyte populations. IL1B^+^ monocytes upregulated the expression of inflammation-related genes (IL1B, VCAN, and FLT1) but did not upregulate the expression of angiogenesis genes, resulting in strong chemotactic activity and a pronounced response to external antigenic stimulation. MNDA^+^ monocytes, characterized by enhanced phagocytic activity, exhibited increased expression of MNDA, LST1, and LIRB2 (Fig. 2B and Additional file 1: Fig. S5D).

To investigate the clinical significance of different MDM subpopulations, we evaluated the top 30 characteristic genes of each subpopulation via the TCGA-GBM/LGG RNA-seq dataset. Strikingly, we found that the estimated proportion of GPNMB^+^ MDMs independently indicated a worse outcome in GBM patients, whereas ICAM1^+^ MDMs demonstrated a better prognosis (Fig. 2C). Kaplan–Meier survival analysis further revealed that patients with high GPNMB^+^ MDM scores and low ICAM1^+^ MDM scores had significantly worse prognoses than did those with low GPNMB^+^ MDM scores and high ICAM1^+^ MDM scores (Fig. 2D). We next evaluated the polarization states of the MDMs using the MacSpectrum algorithm [74]. A total of 1500 cells were randomly selected. The results revealed that GPNMB^+^ MDMs exhibited the lowest macrophage polarization index (MPI) scores, indicating stronger M2-like polarization. In contrast, the MPI scores of ICAM1^+^ MDMs suggested a tendency toward M1-like polarization (Fig. 2E). The activation-induced macrophage differentiation index (AMDI) shows that GPNMB^+^MDM is in a more differentiated state compared to ICAM1^+^MDM (Additional file 1: Fig. S5E). Analysis of the distribution of the GPNMB^+^ MDM and ICAM1^+^ MDM subpopulations in our single-cell dataset revealed that ICAM1^+^ MDMs were highly enriched in patients who responded to neoadjuvant therapy, whereas the proportion of GPNMB^+^ MDMs was relatively low. In recurrent GBM patients, GPNMB^+^ MDMs were the most abundant, which was correlated with the poor prognosis observed in these patients. Both cell subpopulations were present at lower proportions in LGG, which is consistent with our earlier conclusions (Additional file 1: Fig. S5F). Kyoto Encyclopedia of Genes and Genomes (KEGG) enrichment analysis revealed that both subpopulations were enriched in the HIF-1 signaling pathway (Additional file 1: Fig. S5G). These findings highlight the critical roles of GPNMB^+^ MDMs and ICAM1^+^ MDMs in impacting the prognostic outcomes of GBM patients.

To further investigate the biological functional differences between these two subpopulations, we performed GSEA focusing on the classical functional gene sets of macrophages. The results revealed that GPNMB^+^ MDMs were significantly enriched for functional features related to the hypoxia response and glycolysis, whereas functions related to the inflammatory response, leukocyte migration, the immune response-regulating cell surface receptor signaling pathway, phagocytosis, and lymphocyte activation were notably downregulated. Angiogenesis features were not significantly enriched. Interestingly, ICAM1^+^ MDMs exhibited almost the opposite characteristics. Specifically, ICAM1^+^ MDMs were significantly enriched in functions such as the inflammatory response, leukocyte migration, the immune response-regulating cell surface receptor signaling pathway, angiogenesis, and lymphocyte activation, whereas the hypoxia response and glycolysis were markedly downregulated. Notably, phagocytosis-related features were not significantly enriched (Additional file 1: Fig. S5H–J).

We used flow cytometry (FCM) sorting to isolate primary ICAM1^+^ MDMs and GPNMB^+^ MDMs from the tumor samples of GBM patients. Following previous studies, we classified CD45^+^CD11b^+^ cells in gliomas as monocyte-derived macrophages [75] (Fig. 2F–G). We performed mIHC staining to confirm that HIF-1α is weakly expressed in ICAM1^+^ MDMs, whereas it is highly expressed in GPNMB^+^ MDMs (Fig. 2H). We also conducted FCM to assess the expression of HIF-1α in primary ICAM1^+^ MDMs and GPNMB^+^ MDMs (Fig. 2I). Results showed that ICAM1^+^ MDMs respond less strongly to hypoxic stress than do GPNMB^+^ MDMs. We further conducted experiments to assess the functional characteristics of ICAM1^+^ MDMs and GPNMB^+^ MDMs. Human peripheral blood mononuclear cells (hPBMCs)-derived T cells were co-cultured directly with primary MDMs pretreated with ovalbumin (OVA) to establish a direct co-culture system (Fig. 2J). We subsequently assessed the levels of tumoricidal cytokine secretion (GZMB, IFN-γ), exhaustion (PD-1, TOX), and proliferation (CFSE). The results showed that ICAM1^+^ MDMs were capable of activating T cells, whereas GPNMB^+^ MDMs induced T cell exhaustion (Fig. 2K–M).

These data provide insights into the functional heterogeneity of MDMs in GBM and suggest that ICAM1^+^ MDMs and GPNMB^+^ MDMs influence the GBM immune microenvironment in distinct ways. In conclusion, we have provided a detailed description of the heterogeneity and functional characteristics of MDMs in GBM. For the first time, we have defined the prognostically favorable ICAM1^+^MDM subtype and the poor-prognosis GPNMB^+^MDM subtype, with extensive infiltration of ICAM1^+^MDMs in patients who respond to neoadjuvant combination therapy.

### Co-localization of MDMs and TAFs in the histologically defined pathological niches at spatial resolution

To elucidate the spatial heterogeneity of cell distributions, we classified all tumor cells into different molecular subtypes [40], including neural progenitor-like (NPC-like), mesenchymal-like (MES_1-like and MES_2-like), oligodendrocyte progenitor-like (OPC-like), and astrocyte-like (AC-like) (Additional file 1: Fig. S6A). Unlike previous studies [37], which did not distinguish proliferating tumor cells, we identified this group as a separate subgroup due to its lower gene set scores (Additional file 1: Fig. S6B). This provides a fresh perspective for future research. We further categorized these cells into G1/S and G2/M phases on the basis of cell cycle gene expression (Additional file 1: Fig. S6B). To validate our classification, we projected the subtypes onto a two-dimensional butterfly plot (Additional file 1: Fig. S6C). To gain molecular insights into the distinguishing features of our glioma clusters, we selected gene sets and scored the seven identified subtypes. The results revealed that MES_2-like tumor cells had the highest scores for glycolysis and hypoxia, whereas MES_1-like tumor cells had the highest scores for angiogenesis, apoptosis, and the inflammatory response. Interestingly, both MES-like subpopulations presented consistently higher scores for glycolysis, hypoxia, angiogenesis, apoptosis, and the inflammatory response than the other subtypes did but scored lower for OXPHOS (Additional file 1: Fig. S6D). Notably, we found that the hypoxia score was strongly positively correlated with glycolysis, angiogenesis, apoptosis, and the inflammatory response but was significantly negatively correlated with OXPHOS (Additional file 1: Fig. S6E). Above all, our study provides standardized scores for the classic tumor features of each cell subpopulation and conducts a combined analysis of hypoxic features with other biological characteristics. This approach allows us to gain a clearer understanding of the biological functions of different GBM tumor cell subtypes. Finally, we applied deconvolution analysis to the TCGA-GBM/LGG RNA-seq dataset to evaluate the infiltration proportions of different tumor cell types. We found that both MES-like subtypes and proliferating tumor cells were significantly enriched in the GBM patient groups and IDH wild-type groups, suggesting that these four cell types may serve as indicators of poorer prognosis (Additional file 1: Fig. S6F). Moreover, a strong positive correlation was observed between proliferating tumor cells and MES-like tumor cells, leading to the hypothesis that proliferating tumor cells might have the potential to differentiate into MES-like tumor cells (Additional file 1: Fig. S6G).

**Fig. 3:**
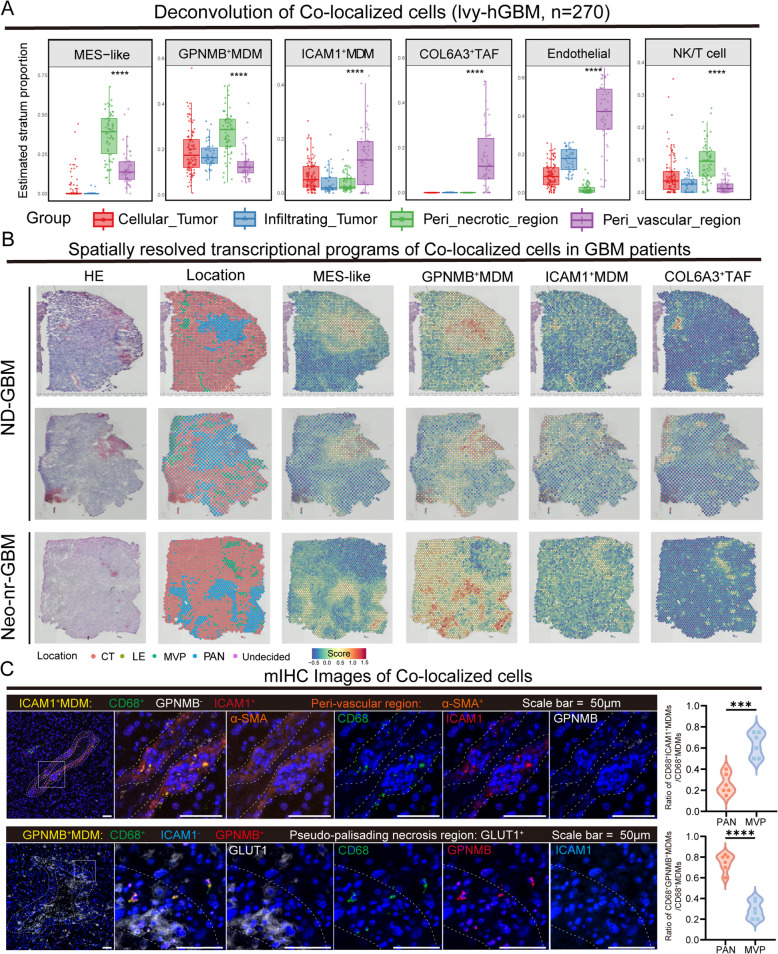
Spatial transcriptomics reveals the co-localization of cell subpopulations in different pathological locations. **A** Box plots showing the estimated proportions of each cell cluster in different histological regions of GBM, obtained through deconvolution of bulk RNA-seq data from the Ivy-GBM cohort (*n* = 270). **B** H&E staining (left) and surface plots (right) showing the distribution of different cell subtypes in newly diagnosed GBM patients and neoadjuvant non-responders. **C** Representative mIHC images of GLUT1, GPNMB, CD68, and ICAM1 in the pseudopalisading necrosis region and α-SMA, CD68, ICAM1, and GPNMB in the peri-vascular region. Box plots (right) displaying the proportions of ICAM1^+^ MDMs and GPNMB^+^ MDMs among all CD68^+^ MDMs in randomly selected high-power fields (HPF, *n* = 6)

Next, we leveraged the Ivy-GBM cohort to integrate the transcriptional signatures of MES-like tumor cells, ICAM1^+^ MDMs, GPNMB^+^ MDMs, COL6A3^+^ TAFs, endothelial cells, and NK/T cells with distinct histological regions of GBM tissues, estimating the relative proportions of each cell cluster (Fig. 3A). Notably, MES-like tumor cells and GPNMB^+^ MDMs were predominantly localized to peri-necrotic regions, whereas ICAM1^+^ MDMs, COL6A3^+^ TAFs, and endothelial cells were enriched in peri-vascular areas.

To further validate these findings, we reanalyzed public GBM spatial transcriptomic datasets [9]. We precisely assigned each spot to one of four distinct pathological regions according to a previous study [76] (LE, leading edge; CT, cellular tumor; MVP, micro-vascular proliferation; PAN, pseudo-palisading cells around necrosis). Additionally, curated gene lists specific to each cellular subtype were applied to classify the spots according to the most representative subtype within each location. The results demonstrated significant co-localization of GPNMB^+^ MDMs and MES-like tumor cells, primarily within the PAN region, both in GBM patients and in patients who did not respond to neoadjuvant therapy. Moreover, ICAM1^+^ MDMs showed notable co-localization with COL6A3^+^ TAFs, which were predominantly localized to the MVP region (Fig. 3B). We further utilized multiplex immunohistochemistry to visualize the distribution of these cell subpopulations. We stained the tumor tissue sections with five markers, α-SMA, GLUT1, CD68, ICAM1, and GPNMB, to exclude the possibility of simultaneous expression of ICAM1 and GPNMB by the same MDMs. The co-staining of GPNMB and the MDM marker CD68 confirmed the proximity of the GPNMB^+^ MDMs to the pseudo-palisading necrosis regions in the GBM tissues (Fig. 3C and Additional file 1: Fig. S6H). Additionally, in the peri-vascular regions identified by α-SMA staining, we observed a significant presence of ICAM1^+^ MDMs marked by ICAM1 and CD68, along with an abundance of COL6A3^+^ TAFs (Fig. 3C and Additional file 1: Fig. S6I). These findings confirm the preferential localization of MES-like tumor cells and GPNMB^+^ MDMs within the PAN niche, while highlighting the enrichment of ICAM1^+^ MDMs and COL6A3^+^ TAFs in the MVP niche. Above all, we expanded upon Neftel et al.’s classification by identifying proliferative tumor cells as a distinct subgroup, indicating their potential to differentiate into MES-like tumor cell subtypes. We also discovered positive correlations between hypoxia and angiogenesis, autophagy, glycolysis, and inflammation, providing new insights into the heterogeneity of GBM tumor cells. Furthermore, we found that MES-like tumor cells and GPNMB^+^MDMs co-localize in the PAN region, whereas COL6A3^+^TAFs and ICAM1^+^MDMs are distributed adjacent to MVP niches for the first time, revealing the unique spatial ecological niches in GBM patients. The co-localization of COL6A3^+^ TAFs and ICAM1^+^ MDMs suggests a potential interaction between these cell populations.

### COL6A3^+^ TAFs mediate the spatial-reprogramming of ICAM1^+^ MDMs into GPNMB^+^ MDMs through TGFβ3 and CSF1

**Fig. 4:**
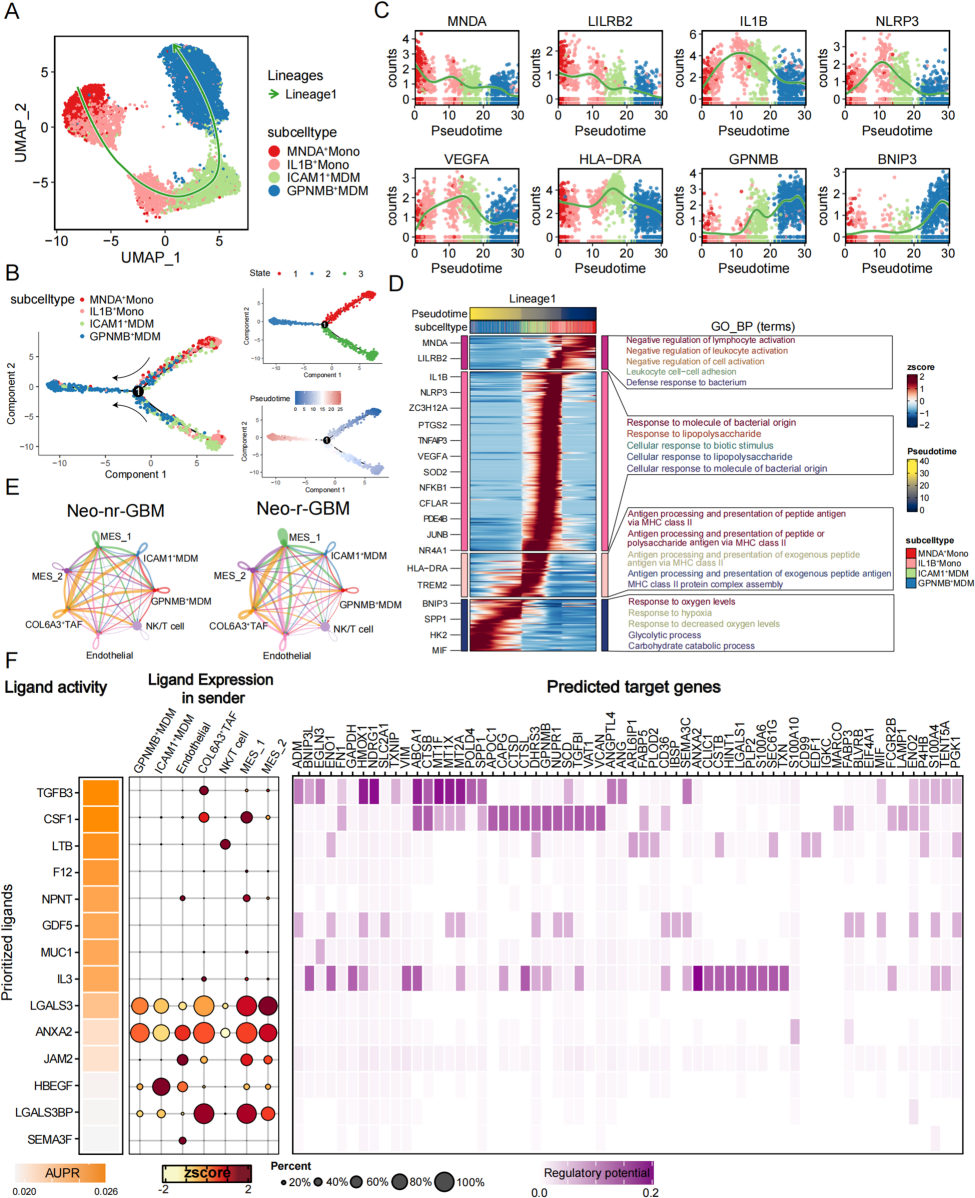
Pseudotime trajectory analysis reveals the differentiation pathways of monocytes/MDMs. **A** UMAP plot showing the monocyte/MDM trajectory derived from Slingshot. **B** Trajectory plots showing the monocyte/MDM trajectory derived from Monocle2, colored based on cell subpopulations (left), cell state (top right), and pseudotime progression (bottom right). **C** Pseudotime projection of transcriptional changes in key genes during differentiation along the trajectory. **D** Heatmap (left) showing the differential gene expression of cells along the differentiation trajectory. On the right, the top five significantly enriched hallmark biological terms at each differentiation stage are displayed. **E** Chord diagram showing the interactions between seven important cell subtypes in the neoadjuvant therapy responders (left) and non-responders (right). **F** Heatmap (left) showing the top-ranked ligands that regulate MDMs by COL6A3^+^ TAFs according to Nichenet. Dot plot (middle) displaying the expression levels of these ligands in different cell subpopulations. Heatmap (right) showing the downstream genes that these ligands activate

One of the fundamental characteristics of GBM is the disruption of the blood–brain barrier (BBB) accompanied by an increased gradient of monocyte chemoattractant proteins (MCPs). This facilitates the detachment of monocytes from the bloodstream, leading to their substantial infiltration into the GBM microenvironment and their differentiation into MDMs [72]. To investigate the dynamic differentiation processes of MDM subtypes at the single-cell level, we extracted MNDA^+^ Mono, IL1B^+^ Mono, ICAM1^+^ MDM, and GPNMB^+^ MDM and performed pseudotime trajectory analysis using the Slingshot algorithm. Our results revealed a differentiation trajectory from monocytes to MDMs, which is consistent with the biological context (Fig. 4A). Interestingly, we observed that the MNDA^+^ Mono resides at the developmental origin, whereas the GPNMB^+^ MDM is positioned at the terminal end of the differentiation axis, with the trajectory indicating a transition from ICAM1^+^ MDM to GPNMB^+^ MDM. To validate the accuracy of this trajectory, we remapped the cells of different subtypes onto a diffusion map dimensionality reduction (DDR) tree via the Monocle 2 algorithm (Fig. 4B). Notably, statistical analyses combining pseudotime and inferred state data revealed two distinct trajectories. However, both trajectories consistently demonstrated the progression from ICAM1^+^ MDMs to GPNMB^+^ MDMs, with both monocytes located at the root of the trajectories. Additionally, we utilized tradeSeq to explore the potential differentially expressed genes and gene expression patterns associated with the differentiation branches involved in MDM state transitions. We observed that the MNDA and LILRB2 genes, characteristic markers of the MNDA^+^ Mono, were predominantly distributed at the initial stage of the trajectory. As pseudotime progressed, the expression of inflammatory genes such as IL1B and NLRP3 significantly increased, indicating the transition of monocytes into an inflammatory state. The expression of angiogenesis genes, such as VEGFA, and antigen-presenting genes, such as HLA-DRA, subsequently increased, whereas the expression of inflammatory genes slightly decreased, suggesting further differentiation of monocytes into MDMs with angiogenic characteristics. At the terminal stage of the trajectory, genes such as GPNMB and BNIP3 were upregulated, whereas the expression of other genes continued to decrease, indicating final differentiation into GPNMB^+^ MDMs (Fig. 4C). As shown in the smoothed heatmap, the highly expressed genes in the early stage of the trajectory within the differentiation branches were functionally enriched in negative regulation of lymphocyte/leukocyte/cell activation and leukocyte cell–cell adhesion, indicating that monocytes in this state are in an immunologically inactive state with strong associations with physical interactions with other cells. In the second stage, the functional characteristics of monocytes reflected a response to external antigenic stimulation, indicating their transition into an inflammatory state. Following this, antigen presentation-related genes were significantly enriched, suggesting that monocytes further differentiated into MDMs to perform antigen presentation and aid in the immune response against the tumor. Finally, due to the highly hypoxic microenvironment of the GBM, MDMs exhibited functional characteristics associated with the hypoxic response, enabling adaptation to this specialized niche (Fig. 4D). Overall, these data provide a detailed depiction of the differentiation trajectory of monocytes/MDMs in GBM, suggesting that ICAM1^+^ MDMs can be reprogrammed into GPNMB^+^ MDMs.

Furthermore, we aimed to investigate the intercellular interaction relationships among co-localized cell subpopulations to further uncover the mechanisms underlying the failure of neoadjuvant combination therapy in GBM. We utilized the CellChat algorithm to uncover intercellular crosstalk relationships. Compared with patients who responded to neoadjuvant combination therapy, stronger interactions were observed among COL6A3^+^ TAF, ICAM1^+^ MDM, and GPNMB^+^ MDM in non-responders, suggesting that COL6A3^+^ TAFs may contribute to therapy resistance by modulating myeloid cells (Fig. 4E). Additionally, we compared the expression of GPNMB^+^ MDM characteristic genes between the high and low COL6A3^+^ TAF infiltration groups in the TCGA-GBM/LGG and CGGA-GBM/LGG cohorts. The results indicated that the presence of COL6A3^+^ TAFs is associated with high infiltration of GPNMB^+^ MDMs, suggesting that COL6A3^+^ TAFs may mediate the spatial-reprogramming of ICAM1^+^ MDMs into GPNMB^+^ MDMs (Additional file 1: Fig. S7A). To identify the key regulators of this process, we employed the Nichenet package to explore the interaction mechanisms between these cell types (Fig. 4F). We designated ICAM1^+^ MDMs as the target cells for the interaction analysis and used the top 50 signature genes of GPNMB^+^ MDMs as the input set of differential genes. Based on prior knowledge from cell–cell interaction signaling network databases, we ultimately identified the cytokines involved in this differentiation process. Results showed that TGFβ3 and CSF1 exhibited the highest levels of ligand activity (Fig. 4F). Since the model of Nichenet also incorporates intracellular signaling information, we further predicted the receptors most strongly associated with these ligands. The results showed that TGFβ3 and CSF1 bind primarily to their respective receptors, TGFBR1, TGFBR2, and CSF1R, on ICAM1^+^ MDMs (Fig. 4F and Additional file 1: Fig. S7B). Thrillingly, we observed that TGFβ3 expression was exclusively restricted to COL6A3^+^ TAFs, suggesting the indispensable role of COL6A3^+^ TAFs in regulating this process (Fig. 4F). Furthermore, spatial analysis of tissue samples confirmed the expression patterns of these ligand-receptor pairs and provided evidence supporting their potential interactions (Additional file 1: Fig. S7C). We next validated the signaling pathways involved in this interaction using CellChat and CellPhoneDB. The results revealed that the TGFβ signaling pathway was active exclusively in COL6A3^+^ TAFs, ICAM1^+^ MDMs, and GPNMB^+^ MDMs. Similarly, the CSF signaling axis was specifically identified between COL6A3^+^ TAFs and MDMs, demonstrating a high likelihood of interaction (Additional file 1: Fig. S7D–F). In summary, based on in silico analyses, we revealed a potentially relevant fibroblast–macrophage interaction. COL6A3^+^ TAFs secrete TGFβ3 and CSF1, which act on TGFBR1/TGFBR2 and CSF1R, respectively, leading to the upregulation of the marker genes of GPNMB^+^ MDMs. These findings suggest that ICAM1^+^ MDMs may undergo reprogramming into GPNMB^+^ MDMs driven by COL6A3^+^ TAFs in response to these signals.

**Fig. 5:**
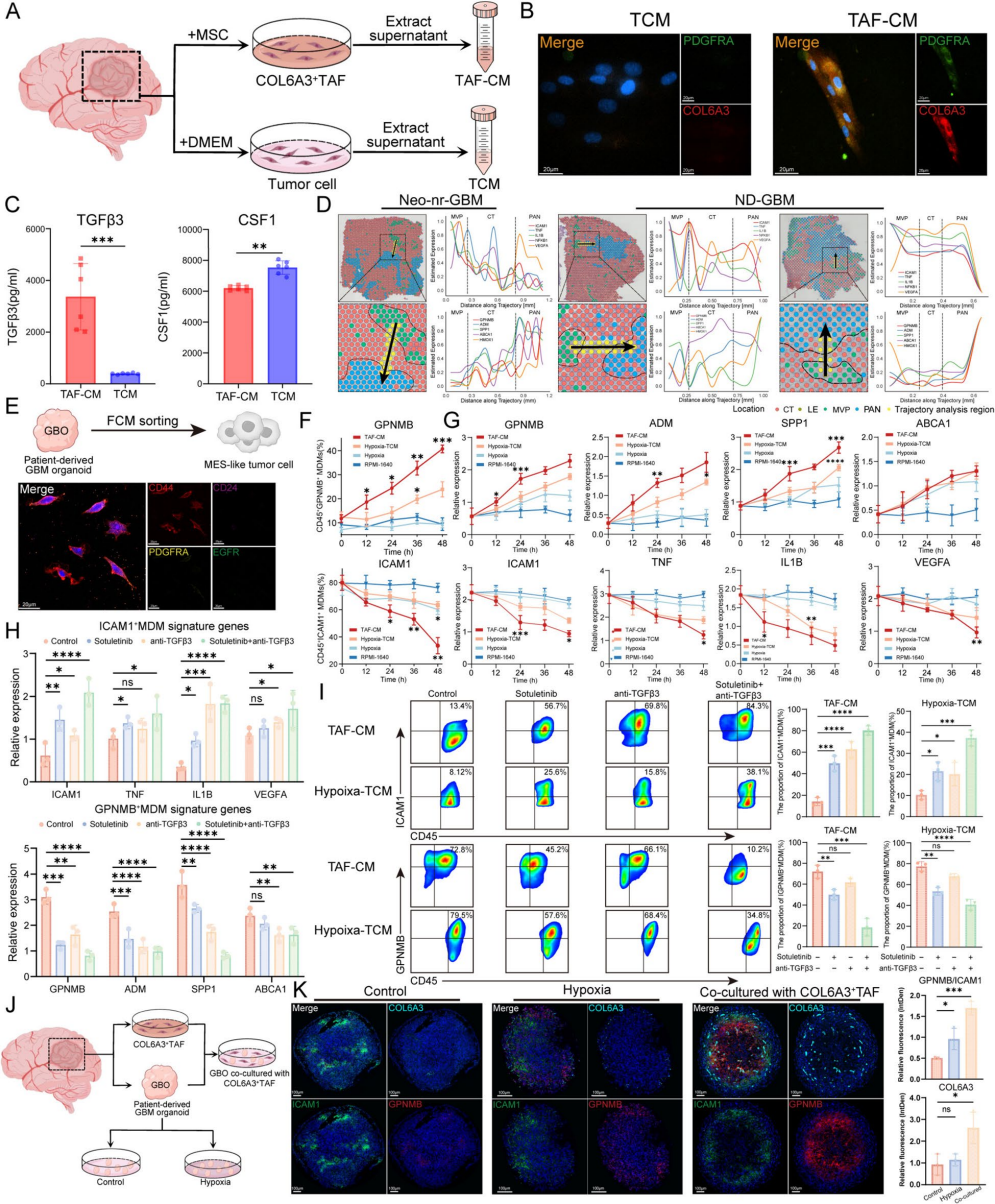
COL6A3^+^ TAFs mediate the spatial-reprogramming of ICAM1^+^ MDMs into GPNMB^+^ MDMs through TGFβ3 and CSF1. **A** Experimental design of the process to obtain TAF-conditioned medium (TAF-CM) and tumor cell-conditioned medium (TCM). **B** Immunofluorescence showing the expression of PDGFRA and COL6A3 in tumor cells and COL6A3^+^ TAFs. **C** Box plots showing the concentrations of TGF-β3 and CSF1 in TAF-CM and TCM. **D** Surface plot displaying the spatial analysis trajectory. Yellow dots represent spatial locations included in the gene gradient analysis. Continuous line plot illustrating the changes in ICAM1^+^ MDM and GPNMB^+^ MDM signature genes from the MVP region through the CT region to the PAN region. **E** Experimental design of sorting of CD44^+^ MES-like tumor cells (above). Representative images showing the expression levels of CD44, CD24, PDGFRA, and EGFR in the sorted cells (below). **F** Line charts showing the temporal changes in the expression of ICAM1 and GPNMB measured via flow cytometry. **G** Line charts showing the temporal expression changes in the signature genes of ICAM1^+^MDM and GPNMB^+^MDM measured by qRT-PCR. **H** Box plot displaying the expression levels of ICAM1^+^ MDM and GPNMB^+^ MDM signature genes under different treatment conditions measured by qRT‒PCR. **I** Representative FCM scatterplots showing the expression levels of ICAM1 and GPNMB under different treatments. **J** Experimental design of establishing patient-derived GBO cultures. **K** Representative mIHC images of COL6A3, ICAM1, and GPNMB (left) and the box plot quantifying the fluorescence intensity (right). The fluorescence intensity of GPNMB was divided by the fluorescence intensity of ICAM1 to assess the extent of MDM reprogramming

To assess whether the spatial-reprogramming of MDMs is influenced by cytokines secreted by COL6A3^+^ TAFs, we conducted extensive experimental validation. We isolated primary cells from GBM patients and cultured them in MSC or DMEM medium (Fig. 5A). The cells cultured in MSC medium expressed high levels of COL6A3^+^ TAF signature genes (COL6A3 and PDGFRA), whereas the tumor cells cultured in DMEM did not express these genes, confirming the accuracy of our isolation method (Fig. 5B). The supernatant from COL6A3^+^ TAFs was collected to obtain TAF-conditioned medium (TAF-CM), while the supernatant from tumor cells was collected to generate tumor cell-conditioned medium (TCM). We then conducted an ELISA to measure the levels of TGFβ3 and CSF1 in the supernatant. Compared with TCM, the supernatant of COL6A3^+^ TAFs contained significantly higher levels of TGFβ3 and slightly lower levels of CSF1, which was consistent with our previous analysis (Fig. 5C). We next developed an in vitro model for MDM differentiation induction (Additional file 1: Fig. S8A). Owing to the high similarity in gene expression profiles, we replaced ICAM1^+^ MDMs with in vitro M1-polarized macrophages. To verify the chemotactic effect of TGFβ3 and CSF1 on MDMs, we added the cell supernatant from COL6A3^+^ TAFs to a transwell chamber seeded with ICAM1^+^ MDMs and introduced sotuletinib (CSF1-R inhibitor) and anti-TGFβ3 to eliminate the activity of the related cytokines (Additional file 1: Fig. S8B). The results showed that these cytokines are crucial in determining the mobilization of MDMs. Combined blockade significantly reduced MDM recruitment to the greatest extent (Additional file 1: Fig. S8B). Next, we performed cell immunofluorescence (IF) to detect the expression levels of ICAM1 and GPNMB. The results revealed that TGFβ3 and CSF1 induced the upregulation of GPNMB expression in ICAM1^+^ MDMs, whereas sotuletinib and anti-TGFβ3 partially inhibited this process (Additional file 1: Fig. S8C). We further confirmed these findings via qRT‒PCR. The results showed that TGFβ3 and CSF1 can induce the transcriptional signature of ICAM1^+^ MDMs to transition into GPNMB^+^ MDMs (Additional file 1: Fig. S8D).

To investigate the gene gradient changes from the MVP region to the PAN region, we performed spatial trajectory analysis. It revealed that ICAM1^+^ MDM marker genes progressively increased, whereas GPNMB^+^ MDM marker genes gradually decreased in both neoadjuvant therapy non-responders and newly diagnosed GBM patients (Fig. 5D). This finding provides evidence supporting the spatial-reprogramming of MDMs. Subsequently, we followed the method described by Gao et al. [28] to isolate a highly purified population of CD44^+^ MES-like tumor cells (Fig. 5E). After the MES-like tumor cells were cultured in a hypoxic environment for 2 days, the supernatant was collected to obtain hypoxia-TCM. Then, we cultured these patient-derived ICAM1^+^ MDMs in TAF-CM, hypoxia-TCM, hypoxia, or RPMI-1640 medium. FCM analysis of GPNMB and ICAM1 expression over time revealed that the expression levels of GPNMB gradually increased under both TAF-CM and hypoxia-TCM conditions, whereas ICAM1 expression showed the opposite trend (Fig. 5F and Additional file 1: Fig. S8E). We also used qPCR to evaluate the temporal changes in the expression of marker genes in GPNMB^+^ MDMs (ADM, GPNMB, HMOX1, and SPP1) and ICAM1^+^ MDMs (TNF, ICAM1, IL1B, and VEGFA) after 0, 12, 24, 36, and 48 h of culture (Fig. 5G). These results indicate that both TAF-CM and hypoxia-TCM can induce the reprogramming of MDMs, with TAF-CM having a more pronounced effect. To further elucidate the critical roles of TGFβ3 and CSF1 in mediating MDM reprogramming, we treated primary ICAM1^+^ MDMs with TAF-CM and separately added sotuletinib and anti-TGFβ3 to block the activity of the respective cytokines. qRT-PCR and FCM analysis showed a significant decrease in the expression of GPNMB^+^ MDM signature genes and a marked increase in ICAM1^+^ MDM signature genes upon cytokine blockade (Fig. 5H–I). Finally, we established patient-derived glioblastoma organoid (GBO) models (Fig. 5J). Under hypoxic culture conditions, the expression ratio of GPNMB to ICAM1 slightly increased. However, co-culture of GBOs with COL6A3^+^ TAFs resulted in a significant increase in this ratio (Fig. 5K). These results suggest that COL6A3^+^ TAFs play a key role in driving the reprogramming of ICAM1^+^ MDMs into GPNMB^+^ MDMs, beyond the effect of hypoxia alone.

Overall, our study demonstrated that COL6A3^+^ TAFs strongly regulate the spatial distribution and functional characteristics of MDMs in GBM. These factors mediate the reprogramming of ICAM1^+^ MDMs into immunosuppressive GPNMB^+^ MDMs through TGFβ3 and CSF1 and modulate the migratory behavior of these cells. Further investigations into the mechanisms underlying this regulation may reveal potential therapeutic targets for treating GBM.

### COL6A3^+^ TAFs enhance vascular fibrosis and lead to T cell exclusion through GPNMB/ITGB5 interplay

**Fig. 6:**
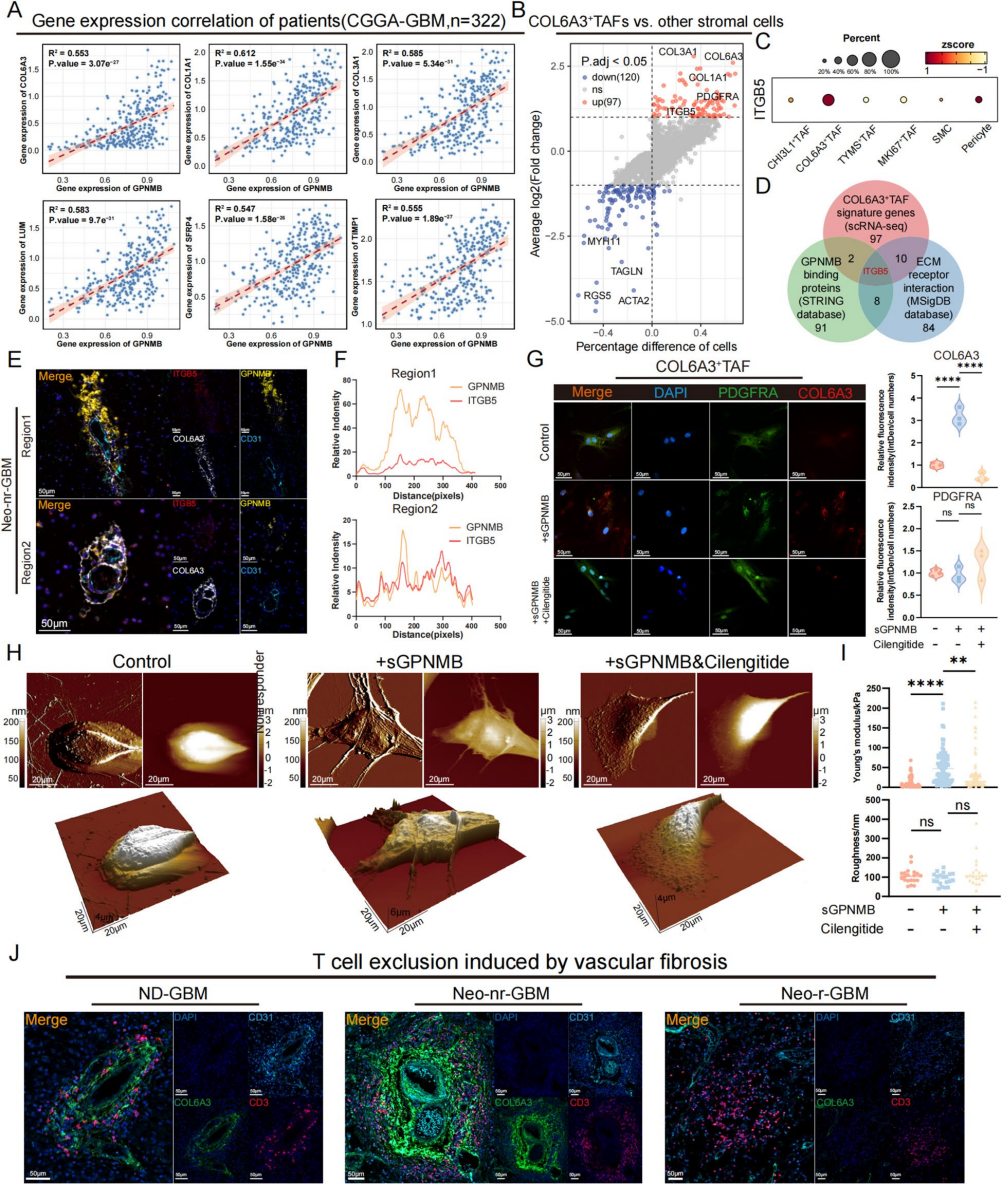
COL6A3^+^ TAFs enhance vascular fibrosis and lead to T cell exclusion through GPNMB/ITGB5 interplay. **A** Pearson correlation analysis showing the associations between GPNMB and fibrosis-related genes in the CGGA-GBM/LGG cohort (*n* = 322). **B** Scatter plot showing the differentially expressed genes between COL6A3^+^ TAFs and other stromal cells. **C** Dot plot showing the expression levels of ITGB5 across different stromal cell subpopulations. **D** Venn diagram showing ITGB5 as a key interacting gene of GPNMB. The data sources include characteristic genes of COL6A3^+^ TAFs from our scRNA-seq dataset, GPNMB-binding proteins from the STRING database, and the ECM receptor interaction gene set from MSigDB. **E** Multiplex immunohistochemistry revealing the co-localization of GPNMB and ITGB5 in non-responder. **G** The line graph quantitatively depicting the fluorescence intensity of GPNMB (yellow) and ITGB5 (red) within the analyzed region. **H** Immunofluorescence staining showing the expression levels of PDGFRA and COL6A3 in different treatment groups (left), and the magnified images were quantified (right). The relative fluorescence intensity was calculated by dividing the total fluorescence intensity of each group by the number of nuclei and normalizing it to that of the control group. The treatment group with only sGPNMB added was the comparison group for statistical significance. **I** Representative atomic force microscopy images showing the cell morphology (above) and three-dimensional distribution of the cell height (below). **J** Box plots quantifying the Young’s modulus and roughness of the cell surface. **K** Representative mIHC images of CD31, COL6A3, and CD3 in newly diagnosed GBM patient (left), neoadjuvant combination therapy non-responder (middle), and neoadjuvant combination therapy responder (right)

We observed that the expression level of GPNMB was highly correlated with the characteristic gene expression of COL6A3^+^ TAFs (Fig. 6A). GPNMB is expressed mainly in macrophages, and its extracellular domain can be cleaved from the cell surface by the protease ADAM10 (A Disintegrin and Metalloproteinase domain-containing protein 10) [77, 78]. The cleaved soluble form can act as a signaling molecule to regulate cellular functional states and contribute to the formation of an immunosuppressive microenvironment [77]. Previous studies have demonstrated that soluble GPNMB (sGPNMB) can bind to the RGD peptide sequence on the surface ligand integrin-αvβ1 of fibroblasts, thereby promoting their fibrotic function [79]. To investigate the ligands of sGPNMB with COL6A3^+^ TAFs, we performed an intersection analysis using three gene sets: the scRNA-seq dataset of COL6A3^+^ TAF signature genes, GPNMB-interacting protein-coding genes from the STRING database, and the ECM–receptor interaction gene set from the MSigDB database. This revealed ITGB5 as the only candidate (Fig. 6B–D). The ligand–receptor pairs between GPNMB^+^ MDMs and COL6A3^+^ TAFs (GPNMB-ITGB5) are generally correlated (Additional file 1: Fig. S9A). Notably, COL6A3^+^ TAFs significantly downregulated the expression of marker genes of myofibroblasts (ACTA2, TAGLN), pericytes (RGS5), and smooth muscle cells (ACTA2, MYH11, TAGLN), indicating that these TAFs represent a distinct group of stromal fibroblasts, unlike those identified in recent studies [80, 81] (Fig. 6B). To demonstrate the potential interaction between the GPNMB and ITGB5, we analyzed their spatial distribution. mIHC demonstrated strong spatial co-localization of GPNMB and ITGB5 around blood vessels, while abnormal fibrotic vasculature was also observed, supporting the possibility of their potential interaction (Fig. 6E–F and Additional file 1: Fig. S9B).

Cilengitide has been developed as a cyclic peptide drug that specifically blocks the RGD peptide sequence on the αVβ3 and αVβ5 integrins. We performed bulk RNA-seq of primary COL6A3^+^ TAFs under three experimental conditions: untreated controls, exogenous supplementation with sGPNMB, and co-treatment with sGPNMB and cilengitide (Additional file 1: Fig. S9C). The principal component analysis (PCA) results demonstrated the differences between our treatment groups and the consistency within each group (Additional file 1: Fig. S9D). Differential expression analysis and boxplots revealed that ECM-related genes were significantly upregulated following the addition of exogenous sGPNMB (Additional file 1: Fig. S9E–F). GO enrichment analysis revealed that, compared with those in the control and cilengitide-treated groups, the genes whose expression was upregulated in COL6A3^+^ TAFs treated with sGPNMB were significantly enriched in terms related to the ECM (Additional file 1: Fig. S9G). These results indicate that GPNMB enhances the functional characteristics of COL6A3^+^ TAFs, including collagen production and ECM remodeling. Further mechanistic analysis revealed that both KEGG enrichment and GSEA revealed the PI3K‒Akt signaling pathway as the most significantly enriched pathway, suggesting that this interaction may further activate the PI3K-Akt signaling cascade (Additional file 1: Fig. S9H–I).

Next, we employed IF quantitative analysis to assess changes in related protein expression across different treatment groups. A significant increase in COL6A3 protein secretion was observed, and this increase was blocked by cilengitide. However, the expression of PDGFRA, a characteristic gene of COL6A3^+^ TAFs, did not significantly change (Fig. 6G). Atomic force microscopy (AFM) can reflect collagen production in COL6A3^+^ TAFs from a cellular biomechanical perspective by measuring the stiffness of the cell matrix. We further used AFM to assess the matrix stiffness of COL6A3^+^ TAFs under different treatments. The results showed that exogenous sGPNMB significantly increased the Young’s modulus of the cell surface without altering its roughness. Similarly, this effect could be alleviated by cilengitide (Fig. 6H–I). These findings suggest that sGPNMB can induce COL6A3^+^ TAFs to secrete more collagen, thereby increasing the stiffness of the vascular matrix and leading to vascular fibrosis as they are distributed around blood vessels. Finally, we collected tumor tissues from newly diagnosed GBM patients, non-responders to neoadjuvant therapy, and responders to neoadjuvant therapy for multiplex immunohistochemistry. The results showed that the dense COL6A3 collagen around blood vessels tightly encircles T cells, forming a physical barrier that prevents T cells from penetrating the tumor core (Fig. 6J). This is the key reason for the failure of combination therapy. In summary, our study revealed that the GPNMB–ITGB5 interplay mediates the increased collagen production and stiffness of highly expanded COL6A3^+^ TAFs in non-responders to neoadjuvant combination therapy. This excessive collagen deposition around blood vessels leads to vascular fibrosis, forming a fibrotic barrier that prevents T cells from penetrating, thereby compromising therapeutic efficacy.

### High infiltration of COL6A3^+^ TAFs and GPNMB^+^ MDMs is correlated with immunotherapy resistance

**Fig. 7:**
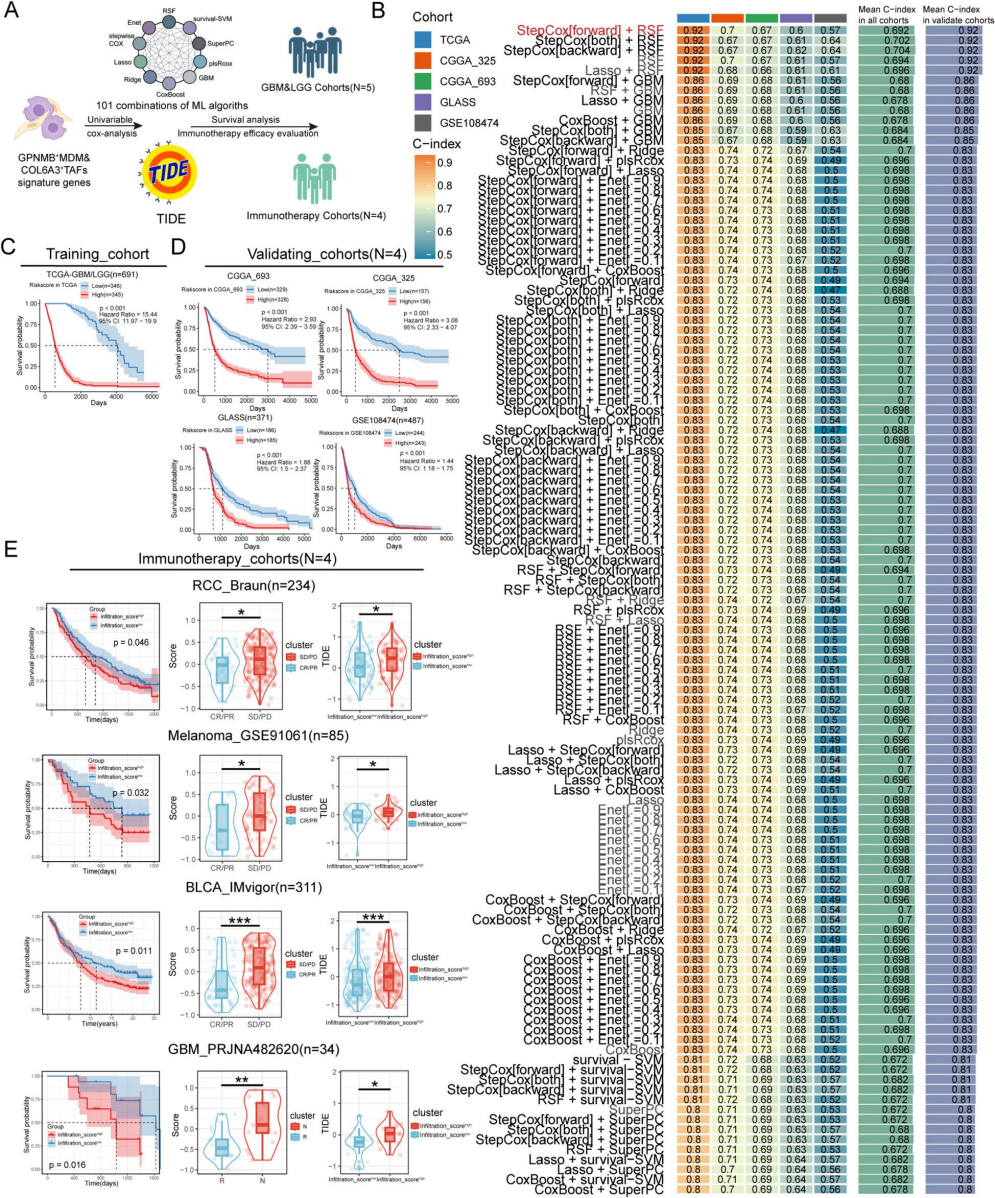
High infiltration of COL6A3^+^ TAFs and GPNMB^+^ MDMs is correlated with immunotherapy resistance. **A** Schematic diagram showing the computational framework for establishing the cell subtype infiltration gene set. **B** Through a tenfold cross-validation framework, a total of 101 combinations of machine learning algorithms were used for the cell subpopulation infiltration gene set. TCGA-GBM/LGG (*n* = 691) was used as the training dataset; CGGA-GBM/LGG (*n* = 693/325), GLASS (*n* = 371), and GSE108474 (*n* = 487) were used as validation datasets. The C-index for each model was calculated based on its application across all datasets. **C** Kaplan–Meier survival curve shows the overall survival of patients in the training cohort, grouped according to the risk scores calculated by the model. **D** Kaplan–Meier survival curve shows the overall survival of patients in the validation cohort, grouped according to the risk scores calculated by the model. **E** Kaplan–Meier survival curves demonstrating the associations between cell infiltration, which is based on GSVA scores, and overall survival of patients across different immunotherapy cohorts (left). Box plots combined with violin plots showing the relationship between cell subtype infiltration scores and treatment response in patients with different therapeutic outcomes (middle), as well as the correlation between infiltration scores and TIDE scores (right)

To develop a prognostic gene set signature, univariate cox regression analysis was used to identify 54 genes from the top 30 marker genes of GPNMB^+^ MDMs and COL6A3^+^ TAFs in the TCGA-GBM/LGG dataset (Additional file 2: Table S5). Ten machine learning algorithms, including RSF, plsRcox, stepwise Cox, CoxBoost, Enet, GBM, Ridge, SuperPC, Lasso, and survival-SVM, were combined based on a tenfold cross-validation to calculate the C-index for the model in the TCGA-GBM/LGG training dataset and four external validation datasets (CGGA_693, CGGA_325, GLASS, and GSE 108474) (Fig. 7A). Ultimately, StepCox (forward) + RSF was considered the best prognostic model, as it had the highest average C-index in the training dataset (Fig. 7B). Since the AUC is another key metric for assessing prognostic models, we conducted a time-dependent ROC curve analysis of the model over a 1- to 5-year period. For five consecutive years, the model showed high AUC scores in the training set and most of the validation sets, further confirming the accuracy of our model (Additional file 1: Fig. S10A–E). The low power of the model in GSE108474 may be attributed to the quality of the microarray data. With the advancement of next-generation sequencing, many machine learning-based prognostic and predictive models have been applied to glioma [82]. We compared the C-index of the model we developed with 95 models retrieved from previous studies. The results indicated that our model outperformed most of the other models in nearly all cohorts (Additional file 1: Fig. S10F). In summary, these results indicate that the prognostic model we developed has high accuracy in predicting patient outcomes.

We further stratified glioma patients into high-risk and low-risk groups based on the median risk score derived from the optimal model and assessed their survival probabilities across each cohort. Notably, patients with higher risk scores exhibited significantly poorer outcomes across all cohorts (Fig. 7C–D). To further investigate the predictive value of the prognostic gene set we developed in immunotherapy cohorts, we evaluated four cohorts that received immunotherapy (RCC_Braun, Melanoma_GSE91061, BLCA_IMvigor, and GBM_PRJNA482620). Survival analysis revealed that individuals with high scores had significantly shorter survival times (Fig. 7E). The tumor immune dysfunction and exclusion (TIDE) score is a predictive biomarker derived from gene expression data and is used to assess the likelihood of immune evasion and response to immunotherapy. Using the TIDE algorithm, we calculated the TIDE scores for each patient in the immunotherapy cohorts. The comparison revealed that clinically responsive patients had significantly lower scores (Fig. 7E). Additionally, based on our gene set, we assessed the infiltration levels of GPNMB^+^MDMs and COL6A3^+^TAFs. We observed that patients with higher infiltration levels had relatively higher TIDE scores than those with lower infiltration levels (Fig. 7E). In summary, high infiltration levels of COL6A3^+^ TAFs and GPNMB^+^ MDMs are correlated with immunotherapy resistance, highlighting their potential role in shaping the immune response and influencing treatment outcomes.

## Discussion

**Fig. 8:**
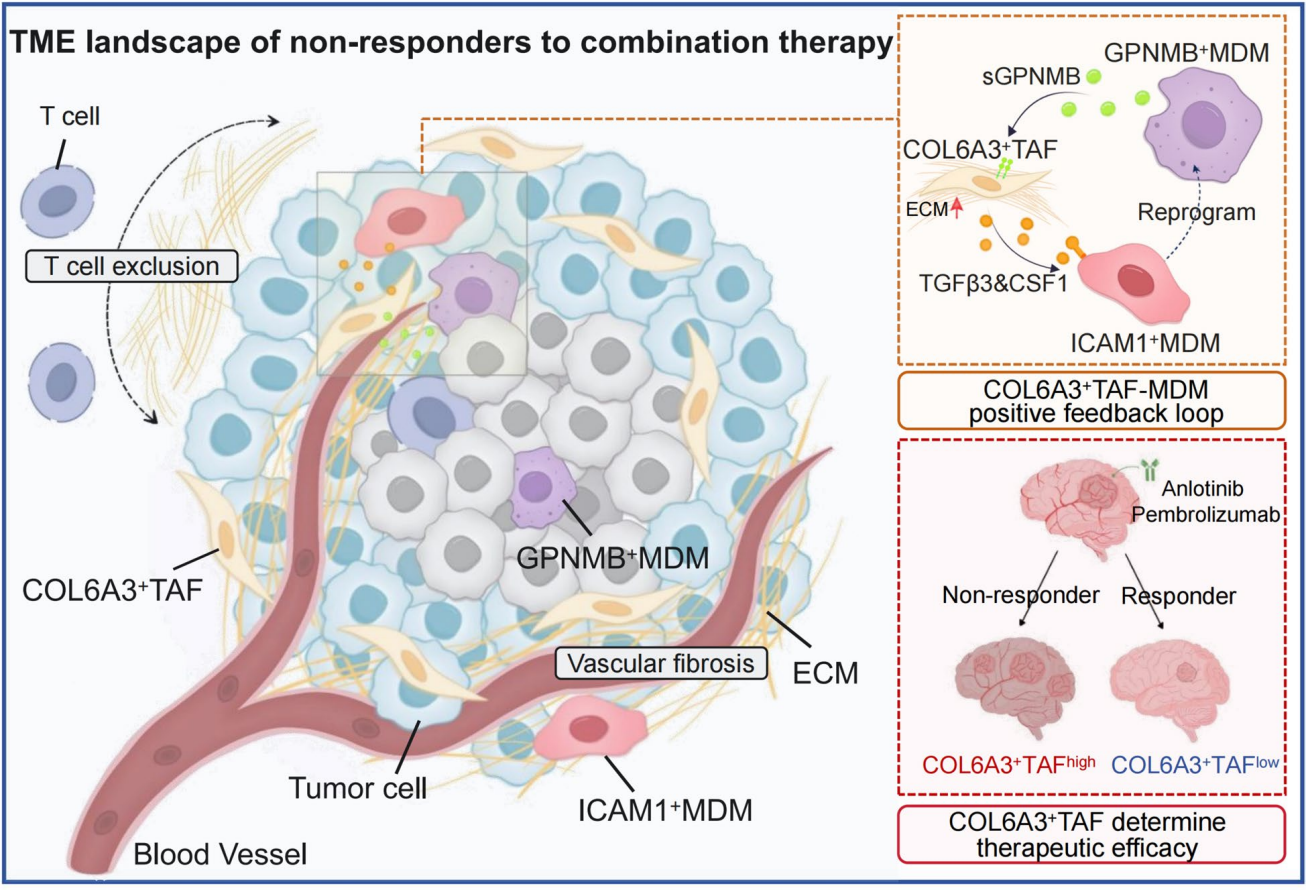
TME landscape of non-responding GBM patients to combination therapy. COL6A3^+^ TAFs and MDMs form a positive feedback loop. Peri-vascular COL6A3^+^ TAFs induce functional and spatial changes in ICAM1^+^ MDMs by secreting TGFβ3 and CSF1. GPNMB^+^ MDMs, which are located in pseudo-palisading necrotic regions, promote COL6A3^+^ TAF-mediated collagen deposition through GPNMB-ITGB5 ligand-receptor interaction, leading to vascular fibrosis and T cell exclusion. The abundance of COL6A3^+^ TAFs can determine the efficacy of combination therapy

Glioblastoma is among the most prevalent and lethal malignant brain tumor and effective therapies are lacking. Many patients with GBM respond poorly to neoadjuvant combination therapy because of the highly immunosuppressive microenvironment, and the molecular mechanisms underlying this phenomenon remain unclear. Therefore, gaining a deeper understanding of the factors underlying the formation of the immunosuppressive microenvironment in GBM and developing entirely new therapeutic targets are particularly important. In this study, we used single-cell transcriptome analysis to construct a comprehensive map of GBM, incorporating neoadjuvant-treated patients into a larger single-cell cohort to minimize statistical bias. Our results indicate that COL6A3^+^ TAFs may play a critical role in driving neoadjuvant combination resistance. These cells shape the immunosuppressive microenvironment and abnormal tumor vasculature by forming a positive feedback interaction loop with MDMs in GBM. Based on this, we provide evidence suggesting that cilengitide offers a promising therapeutic avenue in this context (Fig. 8).

Currently, a variety of immunotherapies have been explored for GBM, including tumor-specific vaccines, dendritic cell therapy, viral therapy, and immune checkpoint blockade. However, none of these treatments has demonstrated significant clinical efficacy in GBM [83–85]. Previous investigations into the failure of these therapies in GBM have focused predominantly on macrophage-mediated mechanisms driving T cell exhaustion [9, 68]. TAFs in GBM represent a key but underexplored cell type, warranting further investigation. Recently, Jain et al. demonstrated that TAFs in GBM can induce the generation of glioma stem cells (GSCs) and promote M2 polarization of macrophages by producing fibronectin containing extra domain A, which interacts with TLR4 on macrophages [32]. Galbo et al. reported that fibronectin derived from TAFs can enhance the invasion and migration of GBM cells and is associated with proneural-to-mesenchymal transition [86]. Zarodniuk et al. revealed that peri-vascular TAFs express various chemokines that recruit tumor-associated macrophages into the tumor microenvironment [87]. Luo et al. reported that CAF-derived LRRC15 in GBM can induce M2 polarization of macrophages and limit the efficacy of PD-1 blockade [81]. In this study, we found that COL6A3^+^ TAFs were significantly more abundant in non-responders than in responders. The CNVs in COL6A3^+^ TAFs are negative, which is consistent with the findings reported by Jain et al. [32]. Our study defines COL6A3 as a characteristic gene of COL6A3^+^ TAFs, with minimal expression in any other cell type within GBM. The expression of COL6A3 is also markedly lower in normal brain tissue, pilocytic astrocytoma, astrocytomas, and oligodendrogliomas compared to glioblastoma. Given their downregulation of the marker genes of myofibroblasts, we consider these cells to be matrix fibroblasts, which is different from the findings of previous studies [80, 81]. Interestingly, we observed that COL6A3^+^ TAFs were primarily localized in areas of GBM microvascular proliferation following neoadjuvant combination therapy, potentially blocking the infiltration of T cells and thereby impairing antitumor immunity. This extensive fiber deposition resulting in fibrotic tumor vasculature may be the cause of further hypoxia in GBM [88]. Moreover, the abnormal vasculature system can hinder drug delivery, thereby limiting the efficacy of antitumor agents in GBM [89]. Collagen can also induce T cell exhaustion [90]. These findings highlight the common mechanism of immune exclusion mediated by COL6A3^+^ TAFs and suggest that COL6A3^+^ TAFs represent a potential therapeutic target for overcoming cancer resistance.

MDMs in GBM are key contributors to tumor progression and the establishment of an immunosuppressive tumor microenvironment [91]. Single-cell RNA sequencing can be used to characterize MDM composition and functional properties across different patients, offering valuable insights into their dynamic interactions and significant influence on tumor progression and treatment response. Our study reports for the first time that there are two distinct subtypes of MDMs in GBM with opposing prognostic gene expression profiles. ICAM1^+^ MDMs are associated with inflammatory and angiogenic programs and exhibit strong T cell activation abilities, whereas GPNMB^+^ MDMs are linked to hypoxic responses and show weaker T cell activation abilities as well as a strong capacity to induce T cell exhaustion. They are respectively localized to the peri-vascular niche and the pseudo-palisading necrotic regions. We also found that hypoxia signaling pathways were activated in both cell types and that GPNMB^+^ MDMs exhibited a greater degree of hypoxia than ICAM1^+^ MDMs did. This finding aligns with previous reports showing that hypoxia plays a key role in regulating the phenotype and spatial distribution of MDMs in GBM while also driving their transition from pro-inflammatory activation to immunosuppressive states [16, 92, 93].

TAFs maintain tight connections with macrophages in the TME [12, 22, 23, 94]. We discovered that COL6A3^+^ TAFs are critical mediators of spatial-reprogramming of MDMs. The strong interactions between COL6A3^+^ TAFs and ICAM1^+^ MDMs via ligand-receptor binding of TGFB3-TGFBR1/TGFBR2 and CSF1-CSF1R induce their reprogramming into an immunosuppressive phenotype and migration, thereby promoting GBM progression. GPNMB is a transmembrane glycoprotein that can be found either on the cell surface or within endosomes and lysosomes [95]. Its membrane-bound portion can be cleaved by metalloproteinases to generate a soluble isoform, which interacts with various receptors to facilitate intercellular communication [96]. High levels of GPNMB expression have been associated with resistance to cancer therapies [77]. GPNMB plays a critical role in tumors. For instance, a study has shown that the macrophage-derived soluble glycoprotein NMB (GPNMB) induces cancer stemness and metastasis via CD44 and IL-33 [97]. Moreover, tumor-associated microglia or macrophages exploit GPNMB to promote tumor growth and alter immune cell infiltration in glioma [98]. GPNMB^high^ macrophages can also secrete GPNMB to promote the PN-MES transition of glioma cells [78]. We identified αVβ5 as the most specifically overexpressed integrin molecule on COL6A3^+^ TAFs and successfully demonstrated that GPNMB^+^MDMs can enhance vascular fibrosis through the GPNMB-ITGB5 interplay. Consequently, we established a positive feedback loop between MDMs and TAFs in GBM patients resistant to neoadjuvant combination therapy. On the one hand, COL6A3^+^ TAFs facilitate the spatial-reprogramming of MDMs; on the other hand, GPNMB^+^ MDMs promote the fibrotic phenotype of COL6A3^+^ TAFs. While our data support the presence of sGPNMB beyond hypoxic areas, we acknowledge that direct evidence for the mechanism by which sGPNMB translocates between tumor compartments is beyond the current scope of our study. Future investigations involving dynamic tracking approaches will be necessary to elucidate the precise transport mechanism involved.

Targeting TAFs has been identified as a promising approach to improve the effectiveness of ICB therapy in preclinical models [30, 99]. Cilengitide is a cyclic RGD mimetic that targets the αVβ3 and αVβ5 integrins [100]. It has been demonstrated to promote vascular normalization in various malignancies (including glioblastoma [101], pancreatic ductal adenocarcinoma [102], non-small cell lung cancer [103], and melanoma [104]), thereby alleviating hypoxia and improving drug delivery. Although cilengitide is now off-patent owing to its failure in clinical trials at the maximum tolerated dose, substantial evidence suggests the potential for repurposing it at low doses in combination with standard therapies [105, 106]. In future studies, we will further explore the potential of low-dose cilengitide in combination therapies while evaluating its efficacy and safety in preclinical and clinical models.

## Conclusions

Our study reveals that COL6A3^+^ TAFs are significantly enriched in patients who did not respond to neoadjuvant combination therapy. We further demonstrate that these cells mediate vascular fibrosis and consequent T cell exclusion by forming a positive feedback loop with MDMs. Moreover, we identify COL6A3 as a signature gene of COL6A3^+^ TAFs. The findings of interaction between COL6A3^+^ TAFs and MDMs provide new avenues for GBM treatment strategies.

## Supplementary Information

Additional file 1: Figures S1–S10 with figure legends. Fig. S1 Experimental workflow. Fig. S2 Generation of a large-scale single-cell transcriptomic atlas of the GBM. Fig. S3 The stromal cells atlas in GBM. Fig. S4 COL6A3^+^ TAF abundance influences clinical outcomes across glioma grades. Fig. S5 Cellular heterogeneity of MDMs in GBM. Fig. S6 Molecular and functional characterization of tumor cells and the spatial distribution of COL6A3^+^ TAF in GBM. Fig. S7 COL6A3^+^ TAFs drive the spatial reprogramming of ICAM1^+^ MDMs to GPNMB^+^ MDMs via TGFβ3 and CSF1. Fig. S8 COL6A3^+^ TAFs drive spatial reprogramming of MDM via CSF1 and TGFβ3 in vitro. Fig. S9 COL6A3^+^ TAFs upregulate ECM-related gene expression through the sGPNMB/ITGB5/PI3K/AKT signaling axis. Fig. S10 Accuracy evaluation of the prognostic model established by machine learning algorithms.

Additional file 2: Tables S1–S6 with legends. Table S1: Clinical characteristics of patients for scRNA-seq. Table S2: Clinical characteristics of patients for organoid. Table S3: Marker genes of cell types, related to Fig. S2 and Fig. S5. Table S4: Marker genes of cell types, related to Fig. 1 and 2. Table S5: The signature genes for prognostic gene set, related to Fig. 7 and Fig. S10. Table S6: Primers used for qRT-PCR, related to Fig. 5.

Additional file 3: Table S7. Genes automatically generated by CIBERSORTx for deconvolution.

Additional file 4: Resource List. The reagent information used in this study.

## Data Availability

The visualization is mainly carried out through the SCP R package [107]. This study did not generate new sequencing data or original code for analysis. All data and code used for analysis in this study are publicly available, and the specific methods for obtaining them are described and cited in the corresponding section of the “Methods”.

## Abbreviations

GBM: Glioblastoma
ICB: Immune checkpoint blockade
TAF: Tumor-associated fibroblasts
TAM: Tumor-associated macrophage
MDM: Monocyte-derived macrophage
PFS: Progression-free survival
OS: Overall survival
ORR: Objective response rate
NSCLC: Non-small cell lung cancer
scRNA-seq: Single-cell RNA sequencing
PDAC: Pancreatic ductal adenocarcinoma
TNBC: Triple-negative breast cancer
THP-1: Human acute monocytic leukemia cell line
mIHC: Multiplex immunohistochemistry
FFPE: Formalin-fixed paraffin-embedded
ECM: Extracellular matrix
LGG: Low-grade glioma
CNV: Copy number variation
CHI3L1: Chitinase-3-like protein 1
SPP1: Secreted phosphoprotein 1
CLU: Clusterin
HLA-DPA1: Major histocompatibility complex, class II, DP alpha 1
CD74: Invariant chain
MDK: Midkine
SMC: Smooth muscle cells
ACTA2: Actin alpha cardiac muscle
TAGLN: Transgelin
MYH11: Myosin heavy chain 11
RGS5: Regulator of G protein signaling 5
PDGFRB: Platelet-derived growth factor receptor beta
CSPG4: Chondroitin sulfate proteoglycan 4
TYMS: Thymidylate synthetase
MKI67: Marker of proliferation Ki-67
COL6A3: Collagen type VI alpha 3
COL3A1: Collagen type III alpha 1
COL1A2: Collagen type I alpha 2
MMP2: Matrix metalloproteinase-2
CTHRC1: Collagen triple helix repeat containing 1
SFRP4: Secreted frizzled-related protein 4
TCGA: The Cancer Genome Atlas
CGGA: Chinese Glioma Genome Atlas
RNA-seq: RNA sequencing
TREM: Triggering receptor expressed on myeloid cells
C1QC: Complement C1q C chain
SELENOP: Selenoprotein P
ICAM1: Intercellular adhesion molecule 1
GPNMB: Glycoprotein NMB
BNIP3: BCL2/adenovirus E1B 19 kDa interacting protein 3
CSTB: Cystatin B
IL1B: Interleukin-1 beta
TNF-α: Tumor necrosis factor-alpha
VEGFA: Vascular endothelial growth factor A
IFIT1: Interferon-induced protein with tetratricopeptide repeats 1
IFIT3: Interferon-induced protein with tetratricopeptide repeats 3
ISG15: Interferon-stimulated gene 15
HSPH1: Heat shock protein HSP 105 kDa
HSPA6: Heat shock protein family A (Hsp70) member 6
BAG3: Bcl-2-associated athanogene 3
VCAN: Versican
FLT1: Fms-related receptor tyrosine kinase 1
MNDA: Myeloid nuclear differentiation antigen
GSVA: Gene set variation analysis
KEGG: Kyoto Encyclopedia of Genes and Genomes
GO: Gene Ontology
OPC-like: Oligodendrocyte progenitor-like
NPC-like: Neural progenitor-like
AC-like: Astrocyte-like
MES-like: Mesenchymal-like
BBB: Blood-brain barrier
GSEA: Gene set enrichment analysis
ADAM10: A disintegrin and metalloproteinase domain-containing protein 10
PDGFRA: Platelet-derived growth factor receptor alpha
FCM: Flow cytometry
TAF-CM: Tumor associated fibroblast-conditional medium
TCM: Tumor cell-conditional medium
GBO: Glioblastoma organoid
hPBMC: Human peripheral blood mononuclear cell
GZMB: Granzyme B
PD-1: Programmed death receptor 1
CFSE: Carboxyfluorescein succinimidyl ester
OVA: Ovalbumin
TIDE: Tumor immune dysfunction and exclusion score
AMDI: Activation-Induced Macrophage Differentiation Index
MPI: Macrophage Polarization Index
