# Supplementary material for: Spatial-reprogramming derived GPNMB+ macrophages interact with COL6A3+ fibroblasts to enhance vascular fibrosis in glioblastoma
Source: Genome Med. 2025 Oct 31;17:136. doi: 10.1186/s13073-025-01553-2 (PMC12577258; doi:10.1186/s13073-025-01553-2)
Supplement: Supplementary file 1 — Additional file 1: Figures S1–S10 with figure legends. Fig. S1 Experimental workflow. Fig. S2 Generation of a large-scale single-cell transcriptomic atlas of the GBM. Fig. S3 The stromal cells atlas in GBM. Fig. S4 COL6A3+ TAF abundance influences clinical outcomes across glioma grades. Fig. S5 Cellular heterogeneity of MDMs in GBM. Fig. S6 Molecular and functional characterization of tumor cells and the spatial distribution of COL6A3+ TAF in GBM. Fig. S7 COL6A3+ TAFs drive the spatial reprogramming of ICAM1+ MDMs to GPNMB+ MDMs via TGFβ3 and CSF1. Fig. S8 COL6A3+ TAFs drive spatial reprogramming of MDM via CSF1 and TGFβ3 in vitro. Fig. S9 COL6A3+ TAFs upregulate ECM-related gene expression through the sGPNMB/ITGB5/PI3K/AKT signaling axis. Fig. S10 Accuracy evaluation of the prognostic model established by machine learning algorithms. [file 13073_2025_1553_MOESM1_ESM.docx]

**Title Page**

**Supplemental information**

**Spatial-reprogramming derived GPNMB^+^ macrophages interact with COL6A3^+^ fibroblasts to enhance vascular fibrosis in glioblastoma**

**Yinfei Du^1,2,3#^, Xinmiao Long^1,2,3#^, Xuetong Li^1,2,3^, Fan Guan ^2,3^ Wei Gao^2,3^, Kun Deng^2,3^, Shiyi Wang^2,3^, Xiang Lin^2,3^, Meng Huang^4^, Xiaoling She^5^, Shuai Chen^1^, Minghua Wu^1,2,3,6*^**

^1^ The Affiliated Cancer Hospital of Xiangya School of Medicine, Central South University/Hunan Cancer Hospital, Central South University, Changsha 410013, Hunan, China

^2^ The Key Laboratory of Carcinogenesis of the Chinese Ministry of Health, The Key Laboratory of Carcinogenesis and Cancer Invasion of the Chinese Ministry of Education, Cancer Research Institute, Central South University, Changsha, 410078, Hunan, China

^3^ FuRong Laboratory, Changsha 410078, Hunan, China

^4^ Department of Neurosurgery, Xiangya Hospital, Central South University, Changsha, 410008, Hunan, China

^5^ Department of Pathology in Second Xiangya Hospital, Central South University, Changsha, 410011 Hunan, China

^6^ Xiangya School of Public Health, Central South University, Changsha, 410013, Hunan, China

^#^These authors contributed equally

*** Correspondence:**

MinghuaWu: wuminghua554@aliyun.com

The Affiliated Cancer Hospital of Xiangya School of Medicine, Central South University/Hunan Cancer Hospital, Central South University, Changsha 410013, Hunan, China

**Supplemental Figure**

**Fig. S1**

**
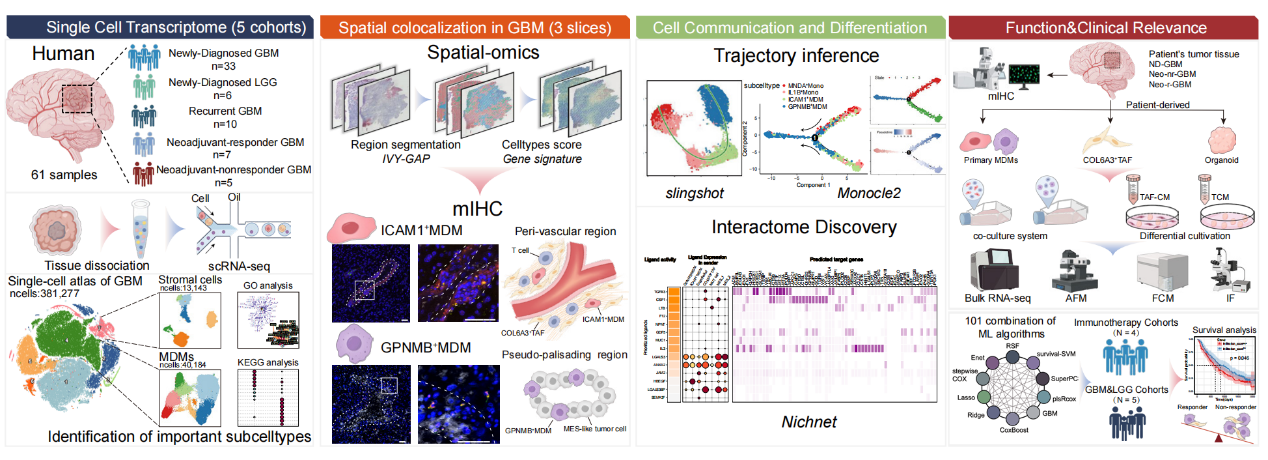
**

**Fig. S1 Experimental workflow.**

**Fig. S2**

**
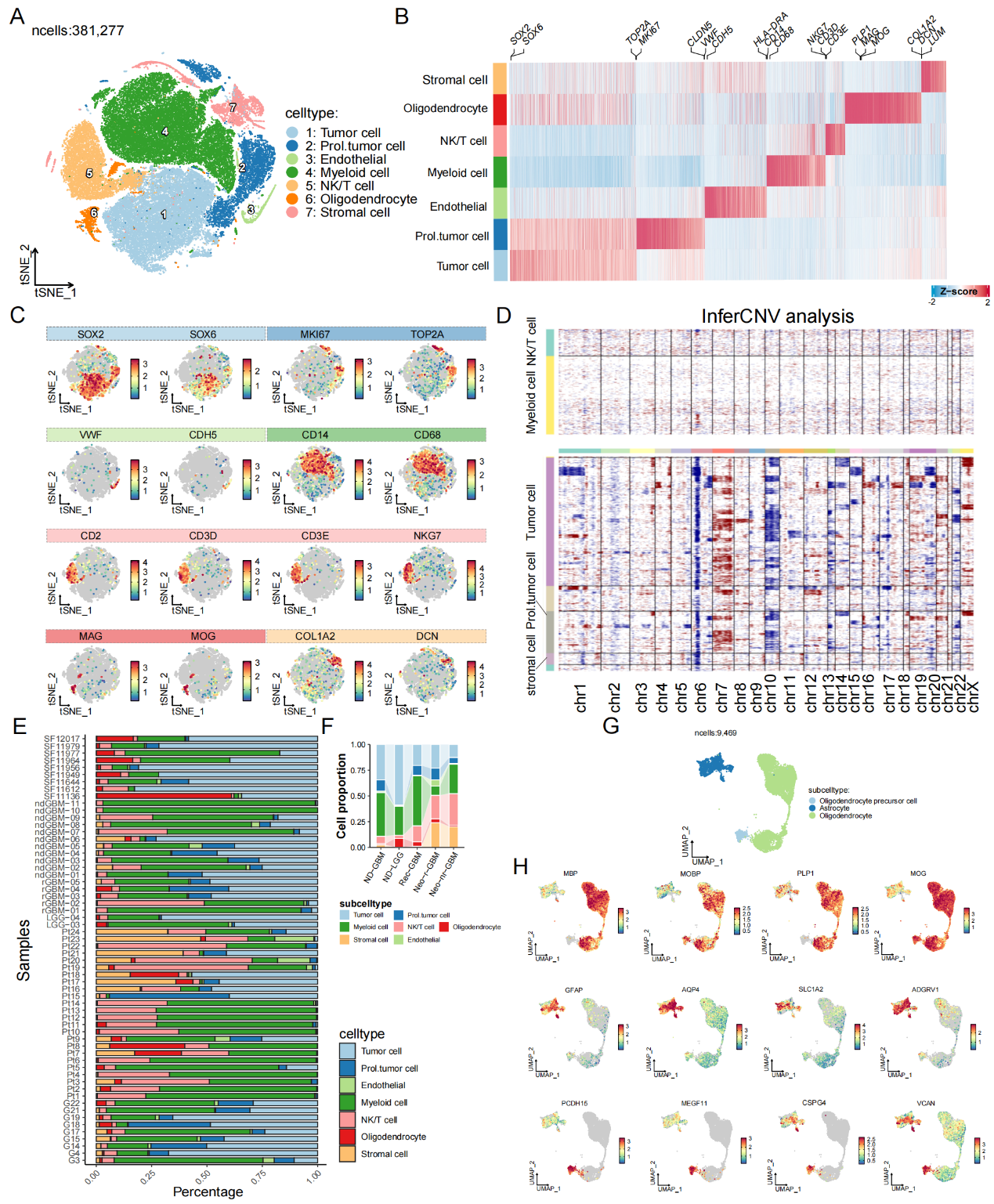
**

**Fig. S2** Generation of a large-scale single-cell transcriptomic atlas of the GBM. **A** UMAP plot showing different celltypes in the GBM microenvironment (n = 381,277 cells). **B** Heatmap displaying the marker genes for each cell type. **C** UMAP feature plots demonstrating the specific gene signatures used for the annotation of cell types. **D** Heatmap illustrating the CNV in tumor cells, proliferating tumor cells, and stromal cells. **E** Continuous bar chart illustrates the proportion of various cell types across different sample classifications. **F** Bar chart illustrates the proportion of various cell types across different patients. **G** UMAP plot showing the subcluster of oligodendrocytes (n = 9,469 cells). **H** UMAP feature plots demonstrating the specific gene signatures applied for subcluster annotation

**Fig. S3**

**
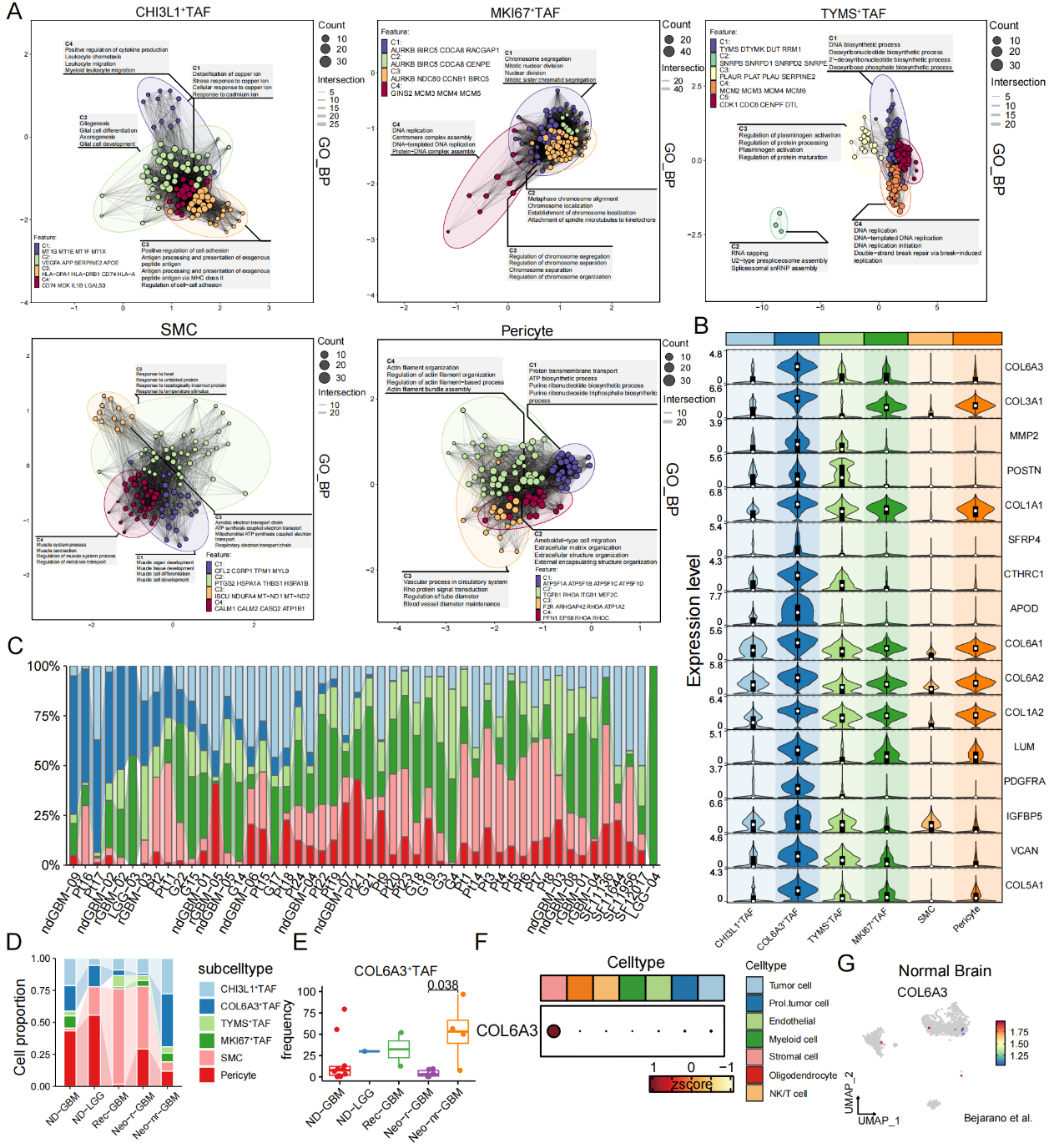
**

**Fig. S3** The stromal cells atlas in GBM. **A** Gene function network diagrams presenting an analysis of the primary functions of genes that are specifically highly expressed across different stromal cell subpopulations. **B** Violin plot displaying the marker genes of COL6A3^+^ TAF. **C** Bar plot showing the differences in proportions of stromal cell subpopulations across patients. **D** Continuous bar chart illustrating the proportion of different subsets of stromal cells across different treatment sample classifications. **E** Box plot showing the proportion of COL6A3^+^ TAF across different sample groups. **F** Dot plot illustrating the expression of COL6A3 in each major cell type. **G** UMAP feature plots showing COL6A3 expression in normal brain tissue.

**Fig. S4**


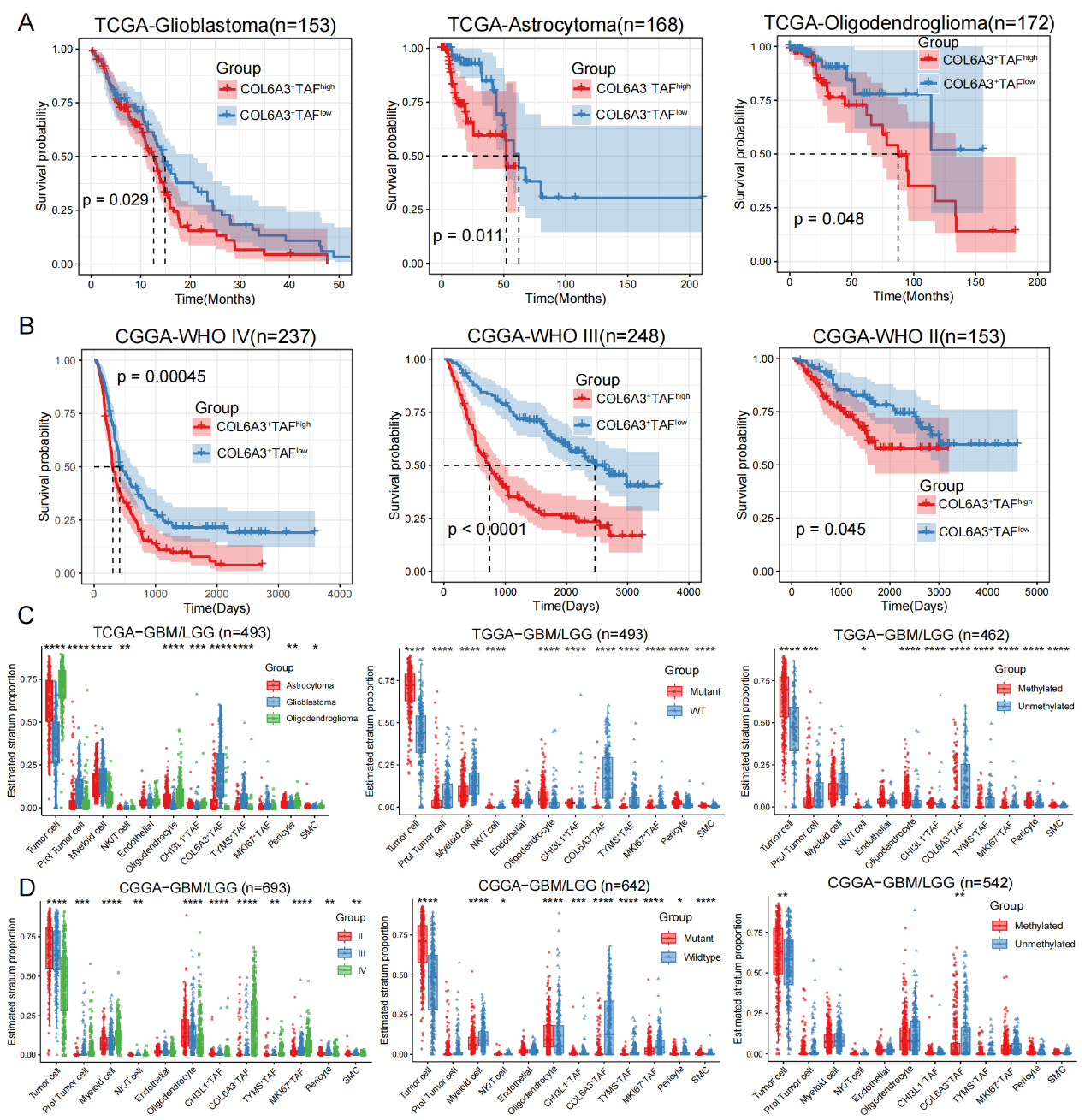


**Fig. S4** COL6A3^+^ TAF abundance influences clinical outcomes across glioma grades. **A** Kaplan–Meier survival curves showing overall survival over time (in months) based on TCGA-GBM/LGG cohorts (Glioblastoma, n = 153; Astrocytoma, n = 168; Oligodendroglioma, n = 172). **B** Kaplan–Meier survival curves showing overall survival over time (in days) based on CGGA-GBM/LGG cohorts (Glioblastoma, n = 237; Astrocytoma, n = 248; Oligodendroglioma, n = 153). **C** Box plots displaying the estimated proportions of different major cell and stromal cell types in the TCGA-GBM/LGG cohorts based on glioma subtype (n = 493, left), IDH mutation status (n = 493, middle) and MGMT promoter methylation (n = 462, right). **D** Box plots displaying the estimated proportions of different major cell and stromal cell types in the CGGA-GBM/LGG cohorts based on 2021 WHO grades (n = 693, left), IDH mutation status (n = 642, middle) and MGMT promoter methylation (n = 542, right).

**Fig. S5**

**
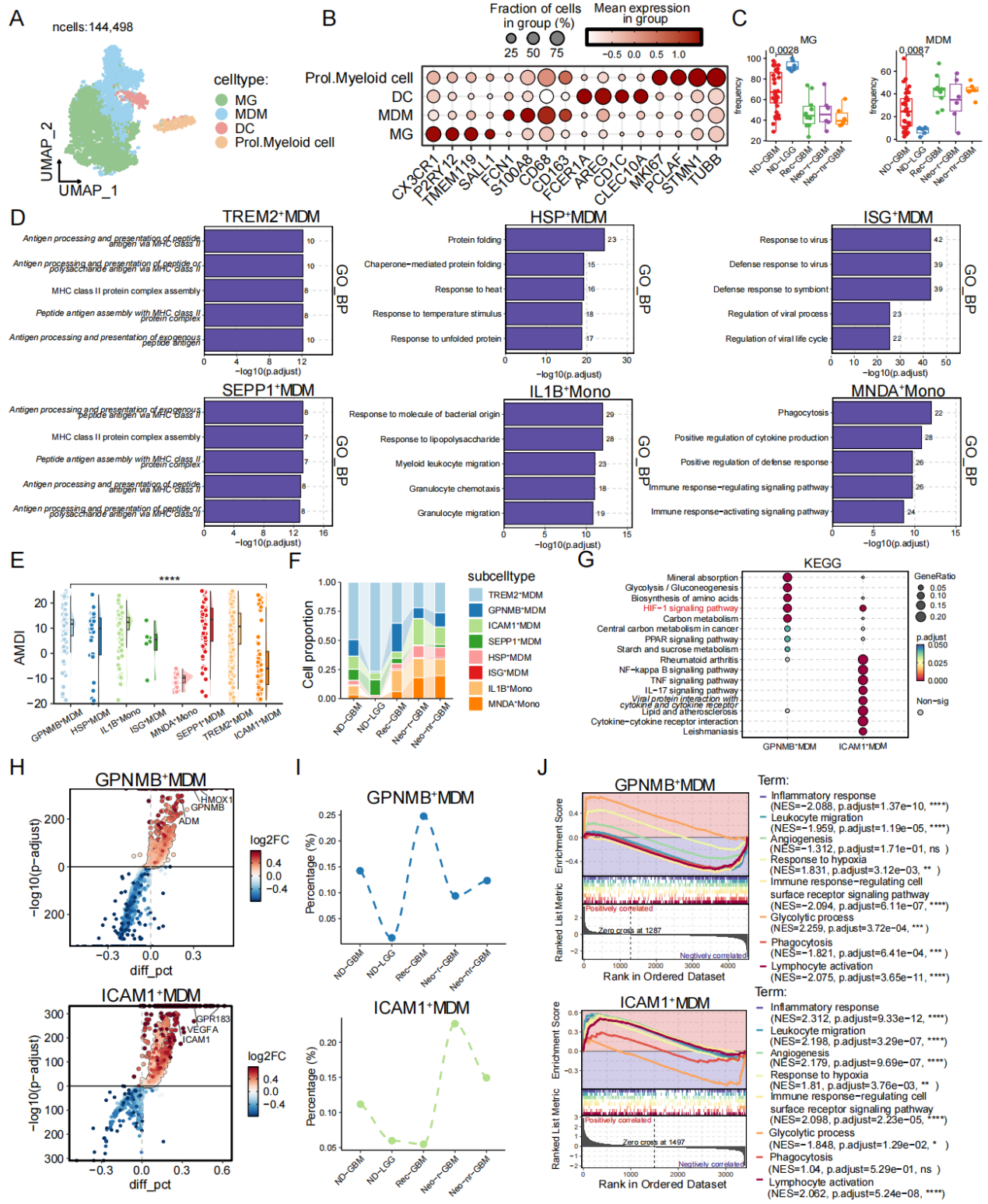
**

**Fig. S5** Cellular heterogeneity of MDMs in GBM. **A** UMAP plot showing different subpopulations of myeloid cells in the GBM microenvironment (n = 144,498 cells). **B** Dot plot showing the marker genes for each subpopulation. **C** Box plot displaying the proportion of MDM and MG cells across different sample groups. **D** Bar chart presenting the GO functional enrichment results of different MDMs subpopulations. **E** Raincloud plots displaying the AMDI scores of different MDM subgroups. **F** Continuous bar chart illustrating the proportion of different subsets of MDMs across different sample classifications. **G** Dot plot showing the top 5 enriched KEGG biological terms for GPNMB^+^MDM and ICAM1^+^MDM. **H** Volcano plot showing the upregulated and downregulated genes in GPNMB^+^MDM (above) and ICAM1^+^MDM (below). **I** Continuous proportion plots showing the infiltration proportions of GPNMB^+^MDM (above) and ICAM1^+^MDM (below) in different patient groups. **J** GSEA plots showing the enrichment of key biological terms related to macrophage function in GPNMB^+^MDM (above) and ICAM1^+^MDM (below).

**Fig. S6**

**
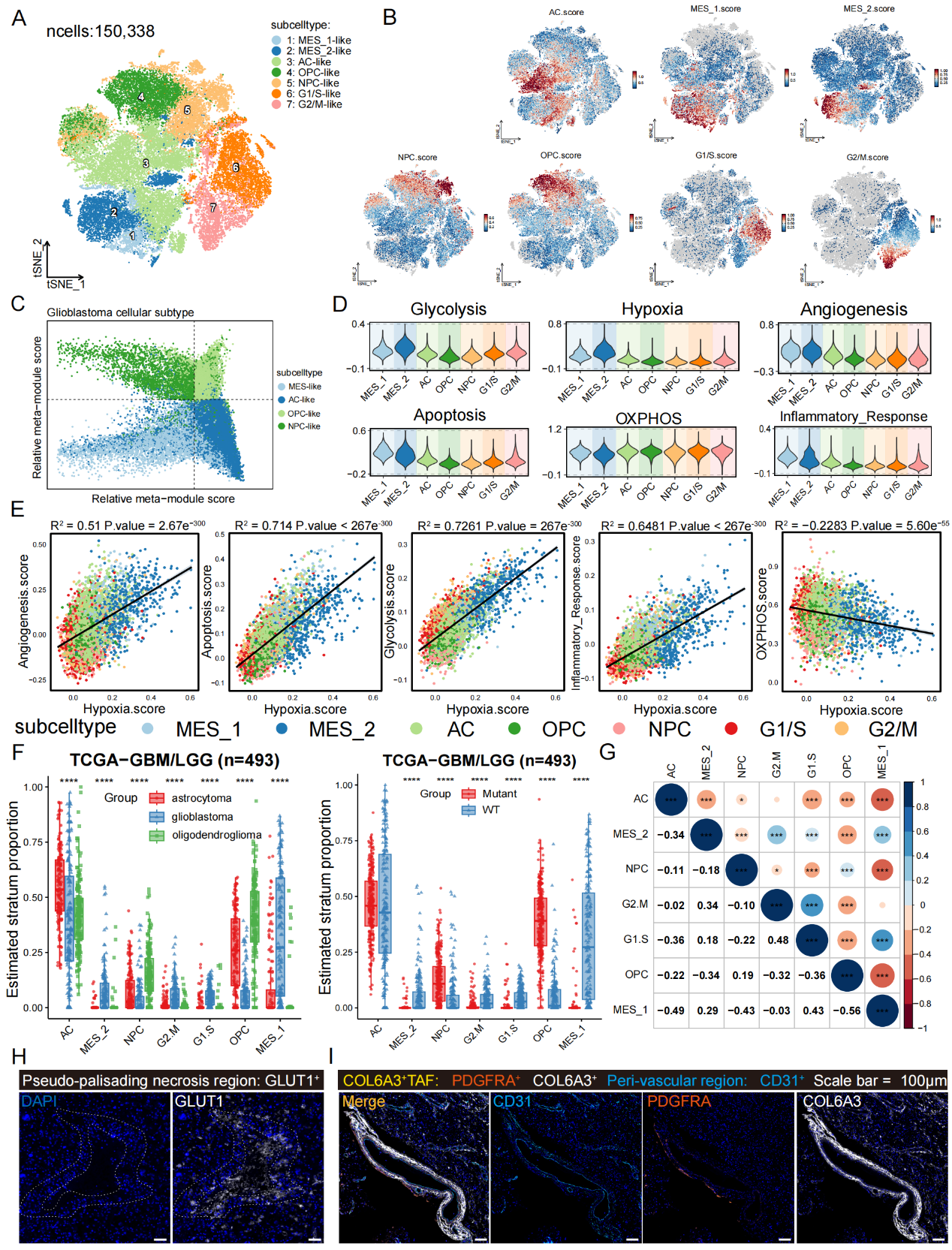
**

**Fig. S6** Molecular and functional characterization of tumor cells and the spatial distribution of COL6A3^+^ TAF in GBM. **A** UMAP plot showing different subpopulations of tumor cells in the GBM microenvironment (n = 150,338 cells). **B** UMAP feature plots demonstrating the scores of different subpopulations of tumor cells. **C** Two-dimensional butterfly plot showing the different tumor cell molecular subtypes classified by Neftel et al and the position of each cell reflecting its relative signature scores. **D** Violin plots demonstrating tumor-associated functional signature scores for different molecular subtypes. **E** Scatterplots demonstrating the correlation between hypoxia and angiogenesis, apoptosis, glycolysis, inflammatory response and oxidative phosphorylation functions. **F** Box plots demonstrating the proportion of different subtypes of tumor cell infiltration in different glioma types (n = 493, left) and IDH mutation status (n = 493, right) in the TCGA-GBM/LGG database. **G** Correlation plot showing the correlation between the number of cells of different tumor cell molecular subtypes. **H** Representative mIHC images of pseudo-palisading necrosis region marked by GLUT1. **I** Representative mIHC images of CD31, PDGFRA and COL6A3 in peri-vascular region.**Fig. S7**

**
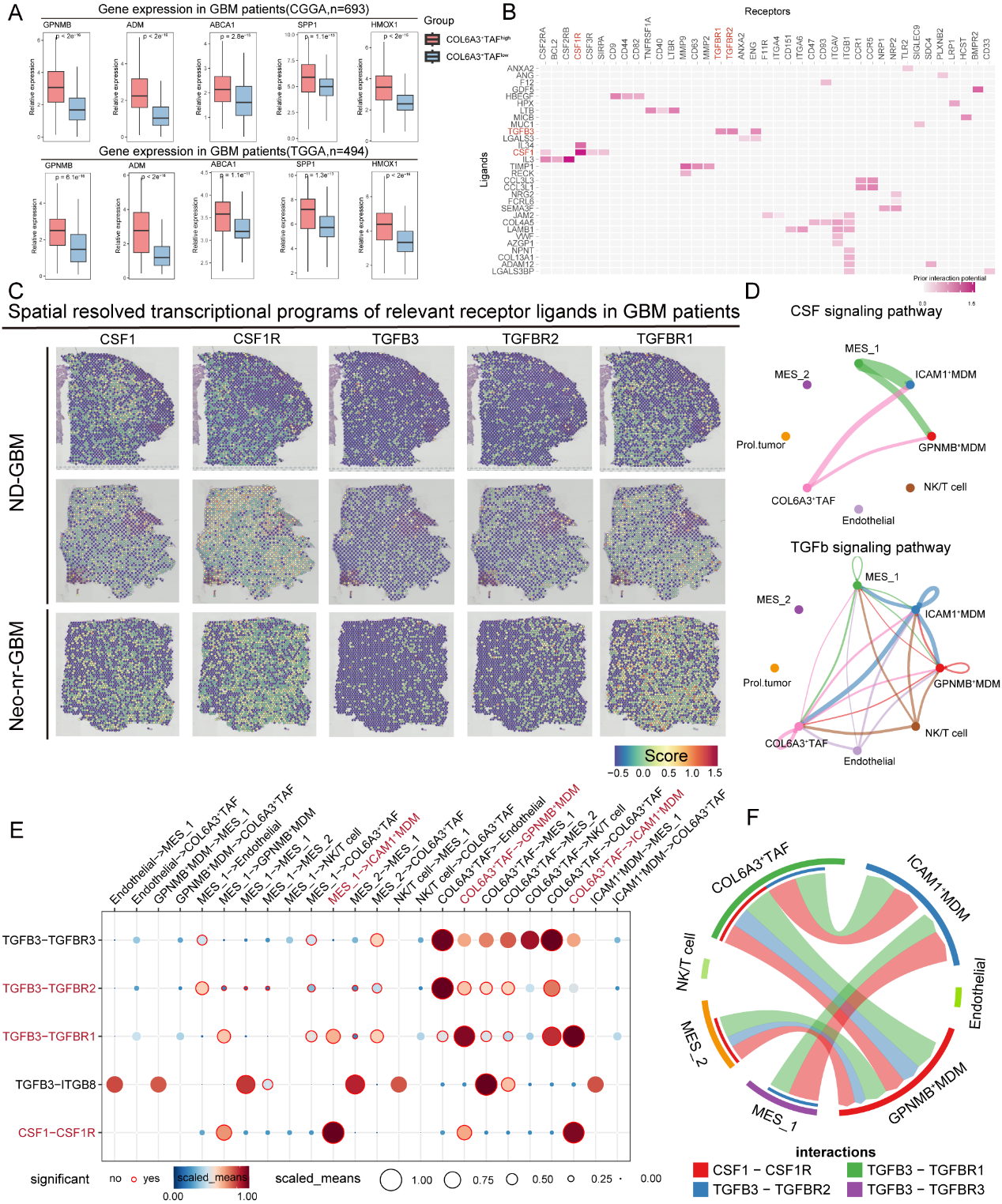
**

**Fig. S7** COL6A3^+^ TAFs drive the spatial reprogramming of ICAM1^+^ MDMs to GPNMB^+^ MDMs via TGFβ3 and CSF1. **A** Box plots showing the expression of GPNMB^+^ MDM signature genes between the COL6A3^+^ TAFs high infiltration group and the COL6A3^+^ TAFs low infiltration group in the CGGA-GBM/LGG (n = 693) and TCGA-GBM/LGG (n = 494) cohorts. **B** Heatmap showing potential reciprocal receptors for TGFβ3 and CSF1. **C** Spatial transcriptome revealing the spatial distribution of TGFβ3 and CSF1 and their receptors in newly-diagnosed GBM patients and neoadjuvant non-responder. **D** Chord diagrams showing that CSF signaling pathway and TGFb signaling pathway are highly enriched between COL6A3^+^TAFs and MDMs. **E** Dot plot showing the results of cell-cell interaction predicted by cellphoneDB. **F** Chord diagram demonstrating the ligand- receptors interactions between different cellular subpopulations.

**Fig. S8**


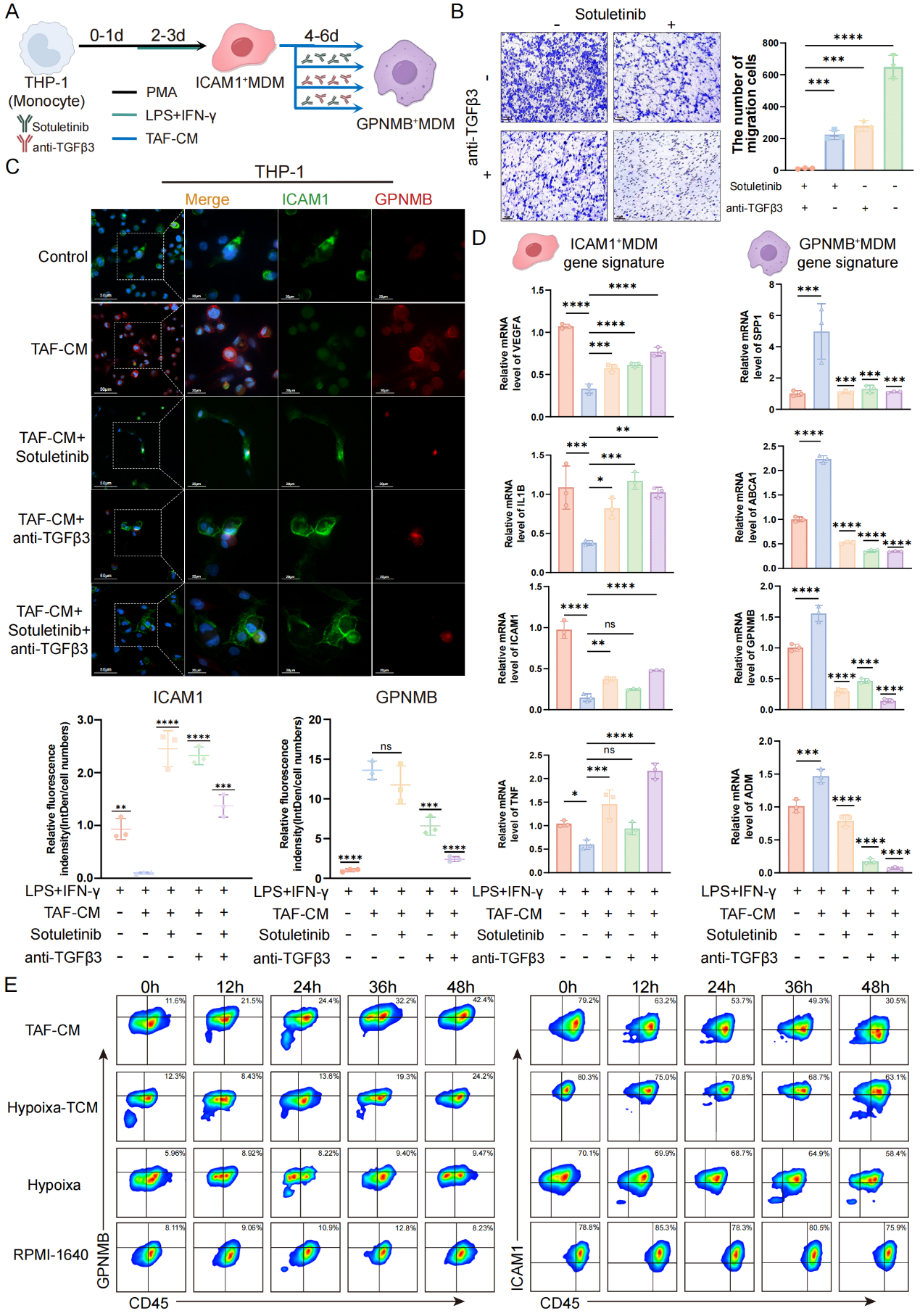


**Fig. S8** COL6A3^+^ TAFs drive spatial reprogramming of MDM via CSF1 and TGFβ3 in vitro. **A** Experimental design of the entire macrophage differentiation induction process. **B** Representative Images (left) showing the results of the transwell assay for chemotactic macrophages, Box plot (right, n = 3) quantifies the number of migrated cells in different treatment groups. **C** Immunofluorescence staining showing the expression levels of ICAM1 and GPNMB in different treatment groups, with quantitative analysis performed on the magnified images. The relative fluorescence intensity was calculated by dividing the total fluorescence intensity of each group by the number of nuclei, and normalizing to the control group. The treatment group with only TAF-CM added was the comparison group for statistical significance. **D** Bar Plots showing the expression of ICAM1^+^MDM marker genes (ICAM1, VEGFA, IL1B, TNF) and GPNMB^+^MDM marker genes (GPNMB, SPP1, ABCA1, ADM) measured by qRT-PCR. The treatment group with only TAF-CM added was the comparison group for statistical significance. **E** Representative FCM scatter plots showing ICAM1 and GPNMB expression levels in primary ICAM1⁺ MDMs under different culture conditions and time points.

**Fig. S9**

**
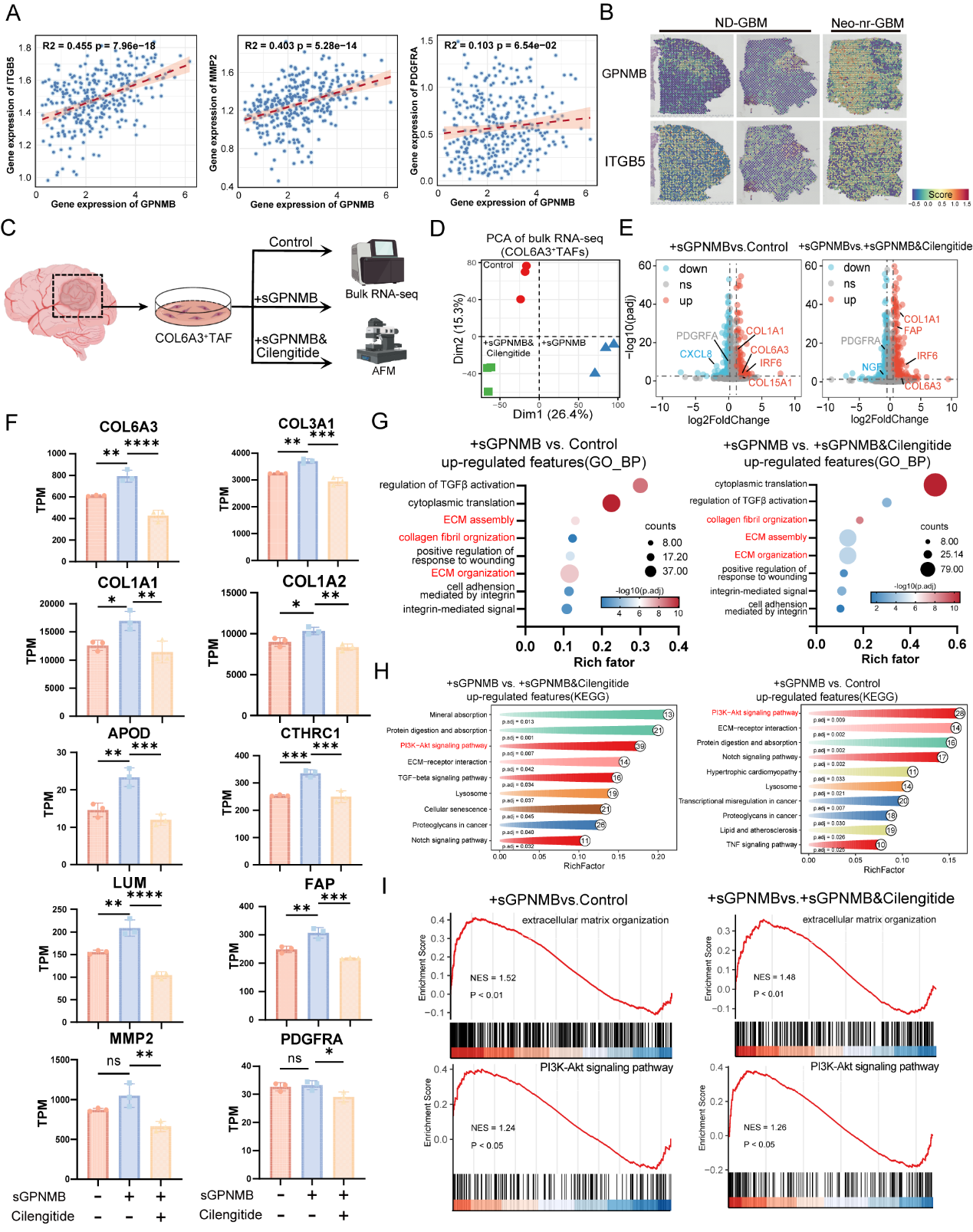
**

**Fig. S9** COL6A3⁺ TAFs upregulate ECM-related gene expression through the sGPNMB/ITGB5/PI3K/AKT signaling axis. **A** Pearson correlation analysis showing the association between GPNMB and fibrosis-related genes in the CGGA-GBM/LGG cohort (n = 322). **B** Spatial transcriptome revealing the spatial distribution of GPNMB and ITGB5 in newly-diagnosed GBM patients and neoadjuvant non-responder. **C** Experimental design of the workflow of COL6A3⁺ TAFs in vitro. **D** PCA plot of bulk RNA-seq data from primary COL6A3^+^ TAFs, COL6A3^+^ TAFs treated with exogenous sGPNMB, and COL6A3^+^ TAFs treated with both sGPNMB and cilengitide.  **E** Volcano plot shows genes that are up-regulated or down-regulated in the COL6A3^+^ TAFs treated with exogenous sGPNMB experimental group compared to the other two groups. **F** Box plot showing the expression of COL6A3^+^ TAF marker genes across the three groups. **G** Dot plots illustrating the significant enrichment of up-regulated genes in GO biological process pathways associated with the exogenous sGPNMB treatment group, compared to the control group (left) and the combined sGPNMB and cilengitide treatment group (right). **H** Bar graphs illustrating the significantly enriched KEGG pathways. The numbers within the circles represent the counts of enriched genes in each term, while different colors denote the classifications of the respective KEGG pathways. **I** GSEA analysis revealing the score of extracellular matrix organization and the PI3K/AKT signaling pathway.

**Fig. S10**

**
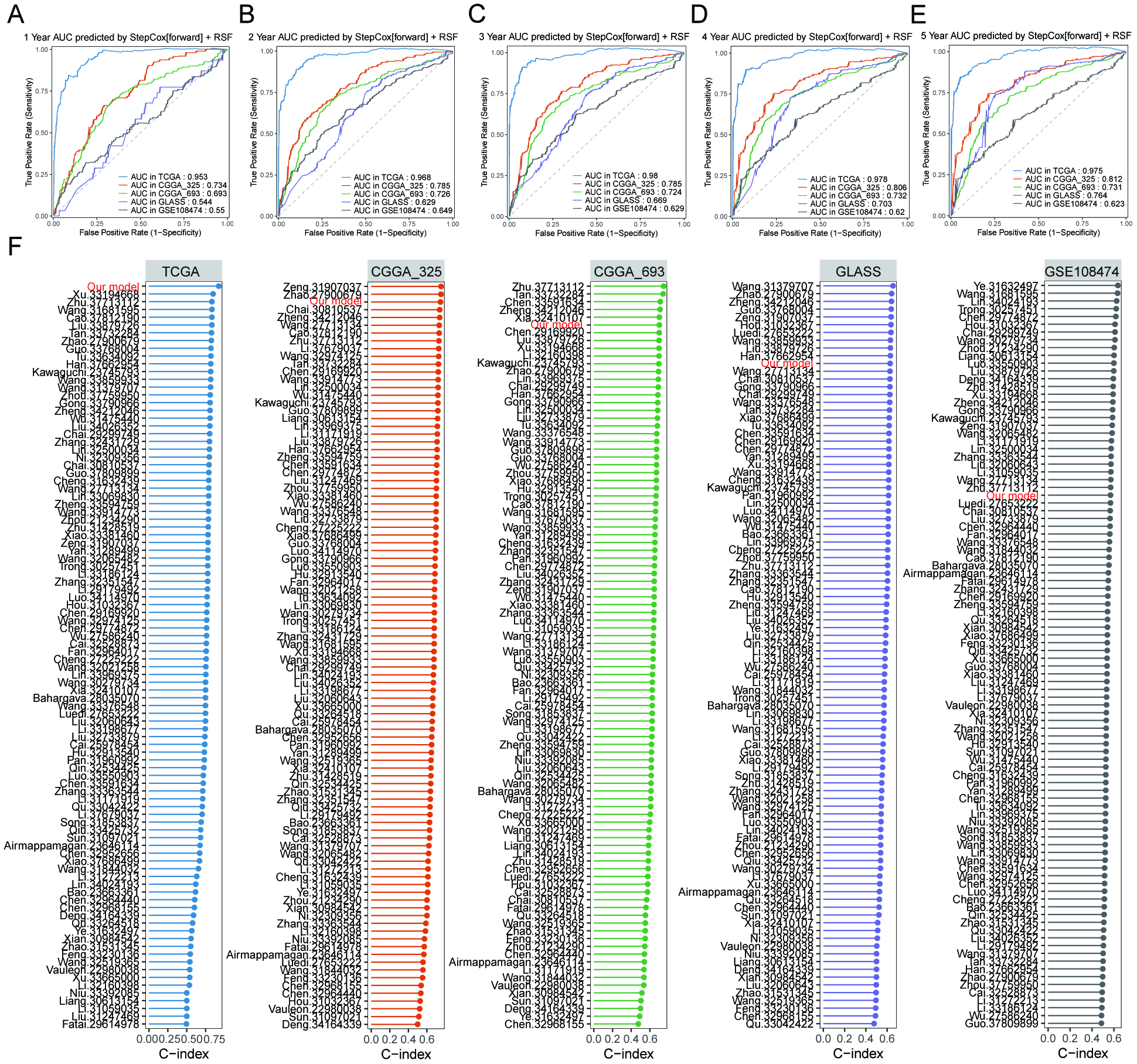
**

**Fig. S10** Accuracy evaluation of the prognostic model established by machine learning algorithms. **A-E** Time-dependent ROC curves for 1-year, 2-year, 3-year, 4-year, and 5-year OS in the training and validation datasets. **F** C-index ranking of the prognostic model we built compared to other models in the training and validation datasets.
