# Supplementary material for: Spatial-reprogramming derived GPNMB+ macrophages interact with COL6A3+ fibroblasts to enhance vascular fibrosis in glioblastoma
Source: Genome Med. 2025 Oct 31;17:136. doi: 10.1186/s13073-025-01553-2 (PMC12577258; doi:10.1186/s13073-025-01553-2)
Supplement: Supplementary file 2 — Additional file 2: Tables S1–S6 with legends. Table S1: Clinical characteristics of patients for scRNA-seq. Table S2: Clinical characteristics of patients for organoid. Table S3: Marker genes of cell types, related to Fig. S2 and Fig. S5. Table S4: Marker genes of cell types, related to Fig. 1 and 2. Table S5: The signature genes for prognostic gene set, related to Fig. 7 and Fig. S10. Table S6: Primers used for qRT-PCR, related to Fig. 5. [file 13073_2025_1553_MOESM2_ESM.docx]

**Supplementary Table**

**Table S1: Clinical characteristics of patients for scRNA-seq**

**Table S2: Clinical characteristics of patients for organoid**

| **Sample** | **Pathology** | **Age** | **Gender** | **MGMT** | **TP53** | **IDH1** | **EGFR** | **1p/19q** | **Treatment** |
| --- | --- | --- | --- | --- | --- | --- | --- | --- | --- |
| 1 | GBM | 67 | Male | NA | Wtp53 | - | + | NA | Newly-diagnosed |
| 2 | GBM | 61 | Female | + | Wtp53 | - | - | -/+ | Newly-diagnosed |
| 3 | GBM | 65 | Male | NA | Mtp53 | - | - | NA | Newly-diagnosed |

**Table S3: Marker genes of cell types, related to Fig. S2 and Fig. S5**

| **Cell Type** | **Marker genes** |
| --- | --- |
| Tumor cell | PTPRZ1,GFAP,SOX2,SOX6,NRCAM,BCAN,NOVA1 |
| Prol.tumor cell | MKI67,TUBB,STMN1,SOX2,SOX6,CENPF |
| Endothelial | CLDN5,VWF,ESAM,PECAM1,CDH5 |
| Myeloid cell | C1QB,HLA-DRA,AIF1,S100A9,C3,CD68,CD14 |
| NK/T cell | CD2,CD3D,CD3E,CD52,NKG7,GZMA,GZMK |
| Oligodendrocyte | PLP1,MAG,MOG,CLDN11,TMEM144 |
| Astrocytes | GFAP, AQP4, SLC1A2, ADGRV1 |
| Oligodendrocyte precursor cells | PCDH15, MEGF11, CSPG4, VCAN |
| Stromal cell | LUM,FN1,RGS5,ACTA2,DCN,COL1A1 |
| MG | CX3CR1,P2RY12,TMEM119,SALL1,FCGR1A |
| MDM | FCN1,S100A8,CD68,CD163,SELENOP |
| DC | FCER1A,AREG,CD1C,CLEC10A |
| Prol.Myeloid cell | MKI67,PCLAF,STMN1,TUBB,CD68 |

**Table S4: Marker genes of cell types, related to Fig. 1 and Fig. 2**

| **Cell Type** | **Marker genes** |
| --- | --- |
| TREM2^+^MDM | SELENOP,APOE,C1QC,TREM2,C3,DAB2,APOC1 |
| GPNMB^+^MDM | GPNMB,MT1G,MIF,BNIP3,CSTB,NUPR1,HMOX1,ADM,  SPP1,PLIN2,LDHA,LGALS1,ERO1A,SCD,ADAM8,VIM  SLC2A1,NDRG1,HK2,SLC6A8,ANGPTL4,FTL,MT1X,MT1H,  MT2A,RNASE1,HILPDA,TPI1,GPI,MXI1 |
| ICAM1^+^MDM | GPR183,IL1B,IER3,NR4A3,REL,CXCL2,PLAUR,ICAM1,  DUSP2,CD83,TNFAIP3,IL1A,KDM6B,OLR1,NFKBIA,PTGS2,  CFLAR,NFKB1,MIR155HG,CCL20,CCL4L2,CXCL3,INSIG1,  EREG,TNF,CXCL8,CCL4,CCL3,ABL2,CCL3L3 |
| SEPP1^+^MDM | SEPP1,ATP5E,GNB2L1,GPX1,ATP5G2,FYB,TCEB2 |
| HSP^+^MDM | HSPA6,BAG3,HSPB1,HSPH1,DNAJB1,HSPE1 |
| ISG^+^MDM | IFIT1,ISG15,IFIT3,XAF1,HERC5,MX1,MX2 |
| IL1B^+^Mono | IL1B,S100A9,S100A8,VCAN,THBS1,FCN1,LYZ,NLRP3 |
| MNDA^+^Mono | S100A8,FCN1,VCAN,MNDA,LST1,LILRB2,CFP,FGR |
| CHI3L1^+^TAF | CHI3L1,SPP1,CLU,HLA-DPA1,CD74 ,MT3,PTPRZ1,MDK |
| COL6A3^+^TAF | COL3A1,COL6A3,COL1A1,SFRP4,CTHRC1,APOD,CYP1B1,  COL6A1,COL6A2,COL1A2,RPS10,TIMP1,IFITM3,VCAN,  RPS8,NNMT,MIF,MFAP2,RPL39,RPL28,LUM,SFRP2,POSTN,  PDGFRA,CXCL6,EEF1G,RPS17,RPL10,RPS12,OGN,SAT1,  IGFBP5 |
| TYMS^+^TAF | TYMS,PCLAF,CLSPN,CCNE2,CENPU,MCM4,CDH2,TK1 |
| MKI67^+^TAF | TOP2A,PTTG1,MKI67,TUBB,PCLAF,CCNB1,CENPU |
| SMC | MYH11,ACTA2,RGS16,NET1,ADIRF,DSTN,TAGLN |
| Pericyte | RGS5,ITGA1,FN1,PDGFRB,THY1,CD248,CYTOR,CSPG4 |

**Table S5: The signature genes for prognostic gene set, related to**

**Fig. 7 and Fig. S10**

| **Gene Symbol** | | | | | |
| --- | --- | --- | --- | --- | --- |
| GPNMB | MT1G | MIF | BNIP3 | CSTB | HMOX1 |
| ADM | SPP1 | PLIN2 | LDHA | LGALS1 | SCD |
| ADAM8 | VIM | SLC2A1 | NDRG1 | HK2 | ANGPTL4 |
| FTL | MT1X | MT1H | MT2A | RNASE1 | TPI1 |
| GPI | MXI1 | COL3A1 | COL6A3 | COL1A1 | SFRP4 |
| CTHRC1 | APOD | CYP1B1 | COL6A1 | COL6A2 | COL1A2 |
| RPS10 | TIMP1 | IFITM3 | VCAN | NNMT | MFAP2 |
| RPL39 | RPL28 | LUM | SFRP2 | POSTN | PDGFRA |
| CXCL6 | EEF1G | RPL10 | RPS12 | SAT1 | IGFBP5 |

**Table S6: Primers used for qRT-PCR, related to Fig. 5**

| **Gene** | **Forward primer (5' to 3')** | **Reverse primer (5' to 3')** |
| --- | --- | --- |
| ICAM1 | ATGCCCAGACATCTGTGTCC | GGGGTCTCTATGCCCAACAA |
| TNF | CCTCTCTCTAATCAGCCCTCTG | GAGGACCTGGGAGTAGATGAG |
| IL1B | ATGATGGCTTATTACAGTGGCAA | GTCGGAGATTCGTAGCTGGA |
| VEGFA | AGGGCAGAATCATCACGAAGT | AGGGTCTCGATTGGATGGCA |
| GPNMB | AAGATTGCCACTTGATGCCG | TCCCTCATGTAAGCAGAAGGTC |
| ADM | ATGAAGCTGGTTTCCGTCG | GACATCCGCAGTTCCCTCTT |
| ABCA1 | ACCCACCCTATGAACAACATGA | GAGTCGGGTAACGGAAACAGG |
| SPP1 | CTCCATTGACTCGAACGACTC | CAGGTCTGCGAAACTTCTTAGAT |
